# Current Trends in the Surgical Treatment of Fibular Fractures: A National Database Study of Intramedullary vs. Plate Fixation Practice Patterns, Complications, and Cost

**DOI:** 10.1155/2024/7506557

**Published:** 2024-07-13

**Authors:** Douglas Zhang, Audrey Litvak, Nicholas Lin, Sean Pirkle, Jason Strelzow, Kelly Hynes

**Affiliations:** ^1^ The University of Chicago Pritzker School of Medicine, Chicago, IL 60637, USA; ^2^ Department of Orthopaedics and Sports Medicine University of Washington, Seattle, WA 98195, USA; ^3^ Department of Orthopaedic Surgery and Rehabilitation Medicine University of Chicago Medicine, Chicago, IL 60637, USA

## Abstract

Existing primary evidence comparing fibular intramedullary fixation (IMF) with traditional plate fixation (PF) for the treatment of distal fibular fractures remains limited by modest sample sizes. Using a large national database, this study aims to compare use rates, fracture patterns, patient characteristics, time to surgery, complication rates, and cost between fibular IMF and PF within the United States. Adults treated with fibular IMF or PF between October 2015 and October 2021 were identified within the PearlDiver Database. The ratio of IMF-treated to PF-treated patients was tracked temporally to compare use rates. Fracture patterns were determined using fracture diagnoses within one-month preceding surgery. Further comparisons of IMF- and PF-treated groups only included patients with at least 12 months of follow-up, and patients with upper tibia or tibia shaft fractures were excluded. An analysis of cohorts matched at a 1 : 4 (IMF: PF) ratio to control for risk factors was performed to compare time to surgery, complication rates (infection, nonunion, malunion, revision, hardware removal, pulmonary embolism, and deep vein thrombosis), and cost. 39329 patients (2294 IMF and 37035 PF) were identified. IMF use trended upwards relative to PF use over time. Tibia and fibula shaft fractures were the most common injuries in IMF patients versus bimalleolar and trimalleolar fractures in PF patients. A higher proportion of IMF patients had open fractures. IMF patients were younger, with higher mean ECI, fewer female patients, and higher rates of CKD. Percutaneous approaches were more common among IMF patients. There were no significant differences in time to surgery or complication rates. IMF was less costly. The popularity of IMF trended upwards across the study period. IMF was used more commonly in injuries involving higher energy trauma and soft tissue disruption. Overall, IMF patients were younger with more comorbidities. When used in similar populations, IMF appears to be a cost-effective alternative to PF.

## 1. Introduction

Globally, ankle fractures are among the most common fractures and are also among the most burdensome in terms of years lived with disability [1–3]. The frequency of these fractures, particularly those resulting from low-energy trauma in older adults, is on the rise [4–10]. The vast majority of ankle fractures involve the distal fibula [4, 7, 11–14] and often occur in conjunction with other fractures [15]. Surgery is indicated for distal fibula fractures that are considered unstable [15]; historically, the most common operative approach in these cases has been open reduction with plate and screw internal fixation [16, 17]. As ankle fractures become more common in older adults and complication rates from standard fixation methods create challenges in higher-risk populations (e.g., diabetics, tobacco-users, and older adults) [18–22], continued investigation of treatment strategies that aim to reduce complications and improve outcomes is warranted.

Intramedullary fibular fixation devices have been shown to provide benefits over plate fixation with respect to complication rates, particularly in patients at higher risk of soft tissue or wound healing complications [23–28]. Intramedullary fibular fixation includes a variety of devices such as k-wires, flexible rods/nails, screws, and fibular-specific intramedullary nails [23, 29, 30]. The purported benefits of fibular intramedullary fixation include the potential for smaller incisions, earlier surgical intervention, earlier weight-bearing, and less associated soft tissue injury, infection rates, and hardware prominence [31–33].

Many studies have already reported promising results from the use of fibular intramedullary devices. A 1986 study on the Inyo Nail, one of the earliest fibular intramedullary nails studied in the literature, reported faster recovery times and better rates of complications, and morbidity for those treated with nail fixation compared to those treated with plate fixation [34]. More recent studies comparing intramedullary fixation to plate fixation of the fibula have also reported benefits in the use of intramedullary devices, including fewer complications, better functional outcomes, faster recovery times, and lower costs [24, 35–38]. However, the results reported within the current body of literature are not in complete agreement, and there remains a need for further research to reach consensus on the optimal implant for distal fibular fractures. Furthermore, little is known about the current utilization rates of intramedullary fibular fixation. Using a nation-wide United States administrative claims database, we aim to compare use rate trends, fracture patterns, patient characteristics, complication rates, time to surgery, and cost between plate fixation (PF) and intramedullary fixation (IMF) of distal fibular fractures. We hypothesize that (1) fibular IMF use is increasing relative to PF use in the United States, that (2) fibular IMF is being used more often in patients with greater comorbidities and higher severity injuries compared to PF, and that (3) fibular IMF allows for similar or lower times to surgery, complication rates, and overall cost compared to PF.

## 2. Materials and Methods

### 2.1. Study Design and Database

A retrospective cohort study was conducted using the PearlDiver Mariner Database (PearlDiver Technologies, Colorado Springs, Colorado, USA), a national insurance claims database that contains de-identified records from nearly 157 million patients. Patients within the database were selected and filtered for this study using International Classification of Diseases, 10th Revision, Clinical Modification and Procedural Coding System (ICD-10-CM and ICD-10-PCS) codes and Current Procedural Terminology (CPT) codes. It is important to note that, while the Mariner Database allows longitudinal retrospective study of complication rates through the tracking of documented billing codes across multiple encounters, it is not possible to track functional or patient reported-outcomes using the database, as these are not typically captured using these codes.

The study population included adult patients (age ≥18 years) treated with plate or intramedullary fixation of the distal fibula between October 2015 and October 2021.

### 2.2. Data Collection

All adult patients treated with plate or intramedullary internal fixation of the fibula were identified using ICD-10-PCS codes for internal fixation (i.e., plate fixation) or intramedullary internal fixation of the fibula (Supplemental Table 1).

#### 2.2.1. Trends in Use Rates and Associated Fracture Patterns

To determine differences in the types of fractures associated with fibular intramedullary and plate fixation, patients with lower leg or ankle fractures that were treated with internal fixation within one month following diagnosis were selected using ICD-10-CM codes for fracture of the lower leg, ankle, tibia, or fibula (Supplemental Table 2). A trend analysis over this period was performed to compare changes in monthly patients treated between the two fixation types.

#### 2.2.2. Comparison of Patient Characteristics

**Figure 1:**
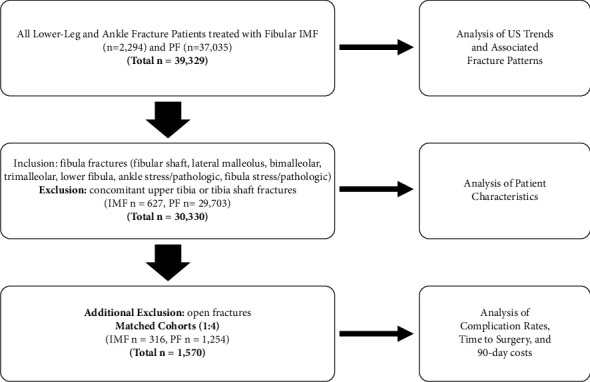
Inclusion and exclusion criteria used to define cohorts for each analysis. In total, 39,329 patients were identified (2,294 IMF and 37,035 PF) and used for analyses of US use rate trends and associated fracture diagnoses. 627 IMF and 29,703 PF patients were further eligible for analysis of patient characteristics. After matching, 316 IMF and 1,254 PF patients were eligible for analysis of complication rates, time to surgery, and 90-day costs.

To determine differences in patient characteristics, 90-day total costs, complication rates, and time to surgery between fixation types, only patients with documented fibula fractures who could be tracked for at least 12 months following surgery were included for analysis, and any patients with concomitant upper tibia or tibia shaft fractures were excluded to ensure that only patients with isolated lower leg and ankle injuries were included (Figure 1). Both ankle and fibula shaft fractures were included to capture all distal fibula fractures treated with internal fixation. Data gathered on patient characteristics included age, gender, US region, operative approach (open or percutaneous), diabetes mellitus (DM), chronic kidney disease (CKD), obesity, tobacco use, osteoporosis, and Elixhauser Comorbidity Index (ECI). ECI is a measure of comorbidity burden, designed to predict risks of in-hospital mortality and 30-day readmission [39].

#### 2.2.3. Analysis of Postoperative Complications and Cost Using Matched Cohorts

Patients with open fractures of the lower leg or ankle were additionally excluded in the analysis of cost, time to surgery, and complication rates to optimize the comparability of groups and ensure that only patients with closed distal fibular fractures were being compared between fixation types. Patients from each treatment group were then sampled randomly and matched at a 1 : 4 (IMF: PF) ratio (Figure 1). The matching process was performed using an exact match methodology to control for the following risk factors: age, gender, DM, obesity, tobacco use, CKD, and associated high-energy fracture (i.e., pilon, lower tibia, and trimalleolar fractures). According to exact match methodology, each PF-treated patient was matched to four IMF-treated patients that fulfilled all the exact same matching criteria. Time to surgery was measured in days spanning from the date of the initial diagnosis to the date of index surgery. Complications of interest included infection, nonunion, malunion, revision, hardware removal, pulmonary embolism (PE), and deep vein thrombosis (DVT). Infection, PE, and DVT were assessed within the 90 days following surgery; revision and hardware removal within one year following surgery; and nonunion and malunion between five weeks and one year following surgery. Cases of nonunion or malunion were only counted if patients additionally had a record of radiologic imaging of the lower leg or ankle that preceded any fibula nonunion or malunion diagnosis within the 5-week to 12-month window. Any cases of hardware removal were only counted as such if they were not associated with a revision procedure. Cost analysis was assessed by totaling all costs across a 90-day period starting from the date of surgery.

Comorbidities (e.g., CKD, DM, and tobacco use) were defined using the corresponding ICD-10-CM codes (Supplemental Table 3), and complications were defined using the corresponding ICD-10-CM codes (infection, nonunion, malunion, PE, and DVT) and ICD-10-PCS and CPT codes (revision and hardware removal) (Supplemental Table 4).

### 2.3. Statistical Analysis

Statistical analyses were aimed at comparing IMF to PF, as PF is the standard, most widely used fixation type for distal fibular fractures. To analyze differences between the two fixation types, Pearson's chi-squared test was used for comparison of categorical variables and Student's *T*-test for comparison of continuous variables. Linear least-squares regression was performed to generate a trendline for the trend analysis. All statistical methods, including the matching process, were conducted using R Statistical Software (v4.1.2; R Core Team 2021), and *p*values less than 0.05 were considered statistically significant.

## 3. Results

### 3.1. Trends in Use Rates and Associated Fracture Patterns

**Figure 2:**
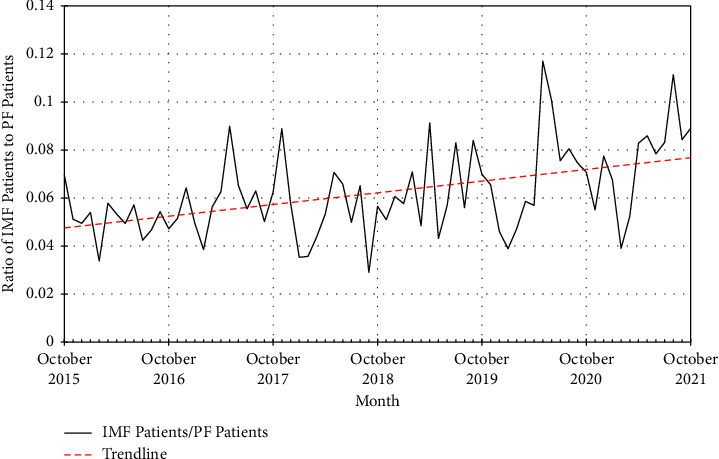
Monthly ratio of patients receiving IMF to patients receiving PF. The rate of IMF use relative to that of PF trended significantly upwards between October 2015 and October 2021 (*R*^2^ = 0.23, *F*[1, 71] = 21.01, *p* < 0.01; *β* = 0.00041, *p* < 0.01).

39,329 patients treated with intramedullary fixation (IMF) or plate fixation (PF) of the fibula were identified: 2,294 received IMF and 37,035 received PF (Figure 1). The monthly ratio of patients receiving IMF compared to patients receiving PF trended significantly upwards across the study period, demonstrating a steady increase in IMF use relative to PF use (Figure 2). Higher proportions of open fractures of the lower leg or ankle were observed among IMF-treated patients than PF-treated patients (28.6% [*n* = 657] vs. 15.9% [*n* = 5,907], *p* < 0.001). The most prevalent fracture diagnoses among IMF-treated patients were fibula shaft (64.8% [*n* = 1,487]) and associated tibia shaft (67.4% [*n* = 1,547]) fractures, while the most prevalent fracture diagnoses among PF-treated patients were bimalleolar (38.3% [*n* = 14,173]) and trimalleolar fractures (38.4% [*n* = 14,233]). The proportions of these respective fracture diagnoses, as well as many other associated fracture diagnoses, significantly differed among IMF-treated and PF-treated patients (ps < 0.001) (Table 1).

### 3.2. Comparison of Patient Characteristics

**Figure 3:**
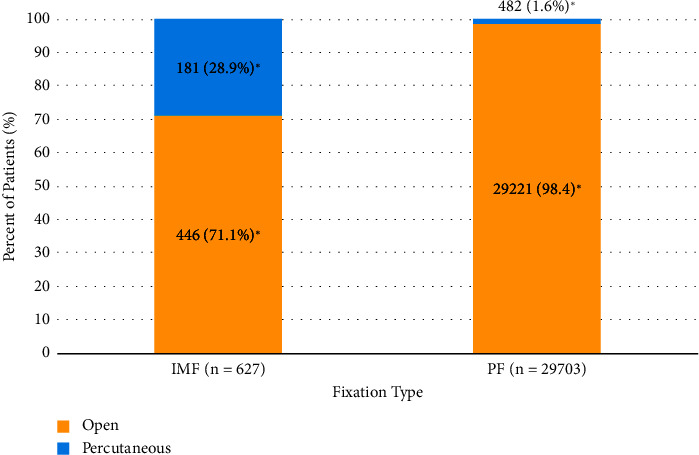
Operative approach (open vs. percutaneous) by fixation type. IMF and PF groups differed significantly with respect to operative approach, with IMF-treated patients more commonly undergoing fixation via percutaneous approaches compared to PF patients, and PF patients more commonly undergoing fixation via open approaches compared to IMF patients (^*∗*^*p* < 0.001).

**Figure 4:**
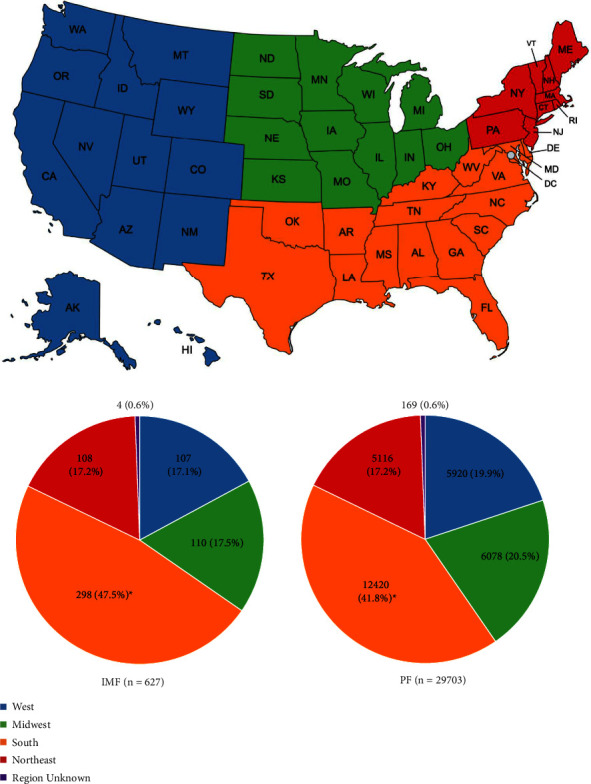
United States regions of IMF and PF patients by fixation type. A significantly higher proportion of IMF patients were treated in the South compared to PF patients (47.5% of IMF patients vs. 41.8% of PF patients, ^*∗*^*p*=0.004). No other significant differences with respect to US regions among patients were observed.

30,330 patients were included in the unmatched analysis of patient characteristics: 627 patients received IMF and 29,703 received PF for treatment of distal fibular fractures (Figure 1). The majority of patients receiving IMF and PF were female; however, a lower proportion of patients receiving IMF were female compared to those receiving PF (64.9% [*n* = 407] vs. 69.7% [*n* = 20,699], *p*=0.010). The median ages of patients receiving IMF and PF were 63.0 (IQR 48–74) and 65.0 (IQR 53–75), respectively, and patients receiving IMF were significantly younger on average compared to those receiving PF (59.4 ± 17.5 years vs. 62.0 ± 15.9 years, *p* < 0.001). Although the majority of patients in both IMF- and PF-treated groups fell into the ages greater than 60 deciles, a significantly lower proportion of patients receiving IMF compared to PF were over 70 years of age (34.8% [*n* = 218] vs. 39.4% [*n* = 11,711], *p*=0.018) and a significantly higher proportion of patients receiving IMF compared to PF were ages 20–29 (7.2% [*n* = 45] vs. 4.5% [*n* = 1,328], *p*=0.001) or 30–39 (8.8% [*n* = 55] vs. 6.6% [*n* = 6.6%], *p*=0.030). The IMF-treated group also had a significantly higher mean Elixhauser Comorbidity Index (ECI) score (7.11 ± 4.77 vs. 6.62 ± 4.34, *p*=0.005) and a significantly higher proportion of chronic kidney disease (24.2% [*n* = 152] vs. 20.1% [*n* = 5,961], *p*=0.010) in comparison to those receiving PF. Higher percentages of diabetes, complicated diabetes, tobacco use, obesity, and osteoporosis were also observed in IMF-treated patients; however, these differences were not significant (Table 2). Additionally, a higher proportion of percutaneous approaches were performed in patients receiving IMF compared to those receiving PF (28.9% [*n* = 181] vs. 1.6% [*n* = 482], *p* < 0.001) (Figure 3). The only significant US regional differences among IMF and PF groups was a significantly higher proportion of IMF patients being from the South compared to PF patients (Figure 4).

### 3.3. Postoperative Complications and Cost between Matched Cohorts

1,570 patients were included in the 1 : 4 (IMF-treated:PF-treated) matched analysis: 316 patients received IMF and 1,254 received PF for closed lower leg fractures involving the distal fibula (Figure 1). No significant differences in relevant patient characteristics were observed between these matched cohorts (Supplemental Table 5). In our analysis of this study group, no significant differences were observed in proportions of postoperative complications of interest, including infection, nonunion, malunion, revision, hardware removal, pulmonary embolism, and deep vein thrombosis (Table 3). Moreover, no significant differences were observed in mean days to surgery (Table 3). However, mean total 90-day cost (in USD) of IMF-treated patients was about $2,600 lower compared to PF-treated patients ($6585.58 ± $10908.50 vs. $9217.24 ± $17772.32, *p*=0.012) (Table 3).

## 4. Discussion

The optimal construct and fixation technique for injuries involving the fibula continues to evolve as surgical techniques and technology improve. Fibular nail technology potentially offers advantages over conventional plate and screw fixation, but this technology remains nascent with only small series reporting outcomes and usage. Our study showed that the rates of patients receiving IMF compared to patients receiving PF have trended upwards. Overall, combined tibia and fibula shaft, lower tibial, and open fractures were more common among IMF-treated patients, while bimalleolar and trimalleolar fractures were more common among PF-treated patients. Intramedullary fixation was also more commonly utilized in those with a greater burden of comorbidities. The high percentage of open and tibia shaft fractures associated with IMF use suggests that IMF is more likely to be used in complex, higher energy injuries, where injury to the fibula may not be the dominant feature of the trauma sustained or when soft tissue concerns necessitate a less invasive approach. Alternatively, it may also be that surgeons treating Weber-C or other high distal fibula fractures (i.e., fibula shaft) may find or believe that an intramedullary implant provides a more stable construct than plating. It is interesting to note that fibular IMF was most used not for isolated ankle fractures, but rather in situations when there was a higher energy injury to the limb, especially in association with tibial shaft fractures. Importantly, despite the different clinical use cases that appear to exist between these two fixation types, the current study does not clearly isolate trends in the treatment of ankle fractures, but rather more broadly identifies how intramedullary fixation of fibula fractures is applied by orthopedic surgeons.

To our knowledge, this study is the first to examine trends in the use rates of fibular intramedullary fixation within the United States. The increasing popularity observed may be explained by the advent of novel intramedullary nail devices for the fibula. The growing amount of literature supporting the purported benefits of these devices, most specifically, fibula-specific intramedullary nails, may be resulting in their increased use as an innovative treatment strategy in high-risk patients, including those with complex injuries [40, 41]. Analogous to our study, which focuses on United States trends, a 2021 study that analyzed trends in the operative treatment strategies for distal fibula fractures in Germany found that the rate of intramedullary nail use for distal fibular fixation increased from 0.1% to 1.0% between 2005 and 2019 [42].

The present study is also the first to our knowledge to comprehensively examine the fracture patterns IMF is associated with treating. Stake et al. investigated the use of fibular intramedullary nails in a hospital where intramedullary nails were primarily indicated for patients with compromised soft tissue, including those with open fractures or preoperative wounds or blisters at the surgical site [43]. In concordance with Stake et al. [43], our study found that IMF was more highly associated with treating open fractures of the lower leg and ankle when compared to PF. In a prospective study of patients with fibular fractures associated with distal tibia and tibia shaft fractures, Stewart et al. stated that the indications for use of a distally locked fibular intramedullary nail include complex injuries or compromised soft tissue [44]. Like the injuries described in that study [44], our study found that complex, high-energy fracture patterns were highly associated with IMF use. Compared to PF-treated patients, IMF-treated patients in our study were much more likely to have associated distal tibia and tibia shaft fractures, the same patterns studied by Stewart et al. [44]. Furthermore, Stewart et al. concluded that using a fibular nail to treat fibula shaft fractures associated with pilon or tibia shaft fractures allowed for minimally invasive approaches and low complication rates while also providing stable fixation [44]. Along with tibia shaft fractures, fibula shaft fractures were seen at a markedly high frequency among IMF-treated patients in our study, and a less invasive percutaneous approach was also more common among IMF-treated patients in our study. This suggests that the motivations for using an intramedullary nail, as described by Stewart et al. [44], may be common among surgeons utilizing fibular IMF for this complex injury pattern. Of note, the percentage of IMF-treated patients treated via a less invasive percutaneous approach in our study (28.9%) is similar to the 30% of fibular intramedullary nail patients treated with a percutaneous reduction in a series of 10 patients reported by Tonks [45].

The existing literature on the applications of fibular IMF has revolved around its use in high-risk patients, including older adults [37, 46–49] as well as those with multiple comorbidities or risk factors, such as diabetes [48, 50, 51], chronic kidney disease [40], tobacco use [52], obesity [52], and osteoporosis [47]. In these patients, IMF may both minimize soft tissue damage [25, 26] and improve local blood flow control necessary for secondary healing by decreasing disruption to periosteal blood supply and increasing periosteal circulation from canal reaming [53, 54]. In agreement with the current literature, our study found higher percentages of all comorbidities and an overall significantly higher mean Elixhauser Comorbidity Index among IMF-treated patients compared to patients receiving PF for distal fibular fractures. However, the average ECI for both groups were relatively low, and the statistically significant difference of 0.49 between average ECI scores (i.e., on average, an IMF patient had 0.49 more comorbidities than a PF patient) may not be clinically significant. Our study also showed that patients receiving IMF for distal fibular fractures were significantly younger on average than patients receiving PF even after considering a narrower subset of associated fracture patterns (i.e., excluding concomitant tibia shaft and upper tibia fractures). Since younger patients are more likely to have high-energy trauma compared to older patients who are more likely to suffer from low-energy fragility injury [4, 6, 55] the comparatively younger age of IMF-treated patients to PF-treated patients may reflect our findings that IMF-treated injuries were more complex, with a higher degree of wound disruption and anatomic associations indicative of higher energy trauma. Despite the IMF group being younger on average than the PF group, most of both cohorts still fell into the ages 60 and over age groups rather than younger age deciles. Patients constituting older age groups are expected to have higher comorbidity burdens, and since comorbidities were assessed between IMF- and PF-treated patients via bivariate analyses (i.e., unadjusted for age), the relatively lower age of IMF-treated patients compared to PF-treated patients may have even deflated the levels comorbidities across the IMF-treated cohort. Hence, it may be the case that older patients receiving IMF compared to older patients receiving PF had even higher average comorbidity burdens than were apparent from our data, which would align with indications for IMF in elderly patients with impaired wound healing [37, 46–49].

While a higher degree of comorbidities, including diabetes, has been discussed as a potential indication for IMF in prior literature [48, 50, 51], CKD is not well-discussed as an indication for fibular IMF; yet, CKD was the only comorbidity found at significantly higher proportions in IMF-treated patients compared to PF-treated patients in our study. CKD is a known risk factor for poor bone quality, both thrombotic and bleeding complications, and impaired wound healing [56–58] and patients with renal disease are often highly comorbid [58], which may simultaneously explain both the higher proportions of CKD and higher mean Elixhauser Comorbidity Index found among IMF-treated patients. Thus, the higher proportion of CKD found among IMF patients in our study may be further evidence that surgeons are turning to IMF as a strategy to minimize the burden of postoperative wound healing and risk of wound-related complications in high-risk patients.

To our knowledge, this is the first study to use matched cohorts to systematically control for differences in risk factors, including comorbidities and severity of injury, when comparing fibular IMF and PF. The use of matching is particularly important given that our findings showed differences in both patient characteristics and fracture diagnoses treated between the IMF and PF cohorts. Given that our findings suggest that there is a preference for choosing IMF over PF in patients with greater comorbidities and injuries involving greater wound disruption and higher energy fracture patterns, it is crucial that we controlled and matched for these factors during our analysis to minimize the possibility that any differences observed are attributable primarily to confounding patient- and fracture-related factors rather than the fixation type chosen. Despite the benefits of fibular IMF reported in the current literature, our study did not find IMF to be associated with significant differences in time to surgery or outcomes except for lower 90-day total costs after adjusting for these risk factors. Overall, we found low percentages of malunion and nonunion in both groups, which agrees with prior and more recent systematic reviews/meta-analyses; however, there were no differences in percentages between groups [27, 38, 59]. Additionally, in accordance with existing evidence, there were no significant differences in PE, DVT, implant removal, or revision between groups [28, 37, 60, 61]. We found that differences in postoperative rates of infection were not statistically significant between cohorts, which disagree with recent meta-analyses showing significantly decreased postoperative infection rates and wound-related complications in IMF-treated patients [28, 62]. Furthermore, higher rates of infection among our matched cohorts than that reported in relevant meta-analyses [27, 28, 62] may be explained by our inclusion of superficial and deep infections in the same analysis as opposed to separate analyses [27], higher average age [63], and prevalence of diabetes of almost 50% [51] among our matched cohorts.

While IMF implants encompass a wide range of devices from simple k-wires and rods to more sophisticated nail designs that could not be controlled for in our cost analysis, our findings align with those of White et al., which did account for upfront implant costs and still showed a decreased overall average cost of healthcare delivery per patient with fibular IMF compared to PF for ankle fracture fixation [24]. Reasons for improved cost-effectiveness of fibular IMF compared to PF may be due to the benefits of lower re-operation rates [28], decreased need for follow-up hardware removal [27], or decreased operative time [24], though these points are debated in the literature [24, 27, 28, 64] and were not shown by our study. Given that we did not account for regional differences with our matching process and that a significantly higher proportion of IMF patients were from the South compared to PF patients, another possibility for the difference in cost observed could be due to regional variations in reimbursement associated with IMF and PF. However, because regional make-up was not included within the matching criteria, our matched analysis is not designed to clarify this possibility. Lastly, we also observed a wide range in costs in both groups, so interpretation of these findings should take that into consideration as well.

### 4.1. Limitations

An inherent limitation of this study is its reliance on the billing codes available for identifying and properly filtering patients within the database [65, 66]. Most limiting was the lack of descriptive billing codes available for identifying intramedullary fibular fixation. While the CPT code 27759 is available for identifying tibia shaft fractures treated with intramedullary implants, there is no such CPT code for the use of intramedullary implants for fixation of fibular fractures. This study instead relied on ICD-10 PCS codes for identifying intramedullary fixation of fibular fractures, which limited our sample to an inpatient sample, prevented us from identifying patients treated prior to October 2015, and may have failed to capture patients whose treatment was documented using only CPT codes. An important consequence of this limitation is that this study may be selecting for patients requiring hospital admission associated with their surgery, which may skew the study sample towards those requiring higher-level care (i.e., sicker or more severely injured patients). To avoid introducing possible bias into any comparative analyses due to this limitation, plate fixation patients were also only identified using ICD-10 PCS codes. Furthermore, the codes available do not distinguish between the various devices that fall under the umbrella of intramedullary fixation devices [65, 66], limiting our ability to interpret our findings more comprehensively. Billing codes are also limited in their level of descriptiveness and clinical relevance. Because much of the recent literature has focused on the use of IMF in treating ankle fractures of the distal fibula, the high percentage of fractures documented as fibula shaft fractures observed in the IMF-treated group was unexpected. This was likely related to the presence of concomitant tibial shaft fractures in these patients. Without access to direct patient data (e.g., patient charts), however, it is not possible to clarify this finding further. It is also important to emphasize that, while longitudinal tracking across extended time-periods is possible using PearlDiver, functional and patient reported-outcomes are not tracked in PearlDiver, as these types of outcome measures are not captured using billing codes. Lack of access to functional and patient reported outcome measures, ordinarily reported as means and ranges, also limits this study's capacity to report on score ranges for important outcome measures, which might otherwise shed light on the consistency of results associated with IMF use compared to PF use. Because of this limitation, this study was limited to reporting primarily rates and percentages (i.e., complication rates). Additionally, given that fibular IMF is much less commonly utilized than PF, surgeon experience is an important factor to consider when studying outcomes, but the database does not provide this information.

Lastly, while we employed a strategic matching process to control for different patient characteristics and the degree of energy associated with a fracture pattern, the overall number of cases made it infeasible to fully include the wide array of fracture diagnoses as separate matching criteria. Furthermore, the inclusion of additional fracture classification analysis (e.g., Weber or Hansen classifications) is not only not possible with the limited data provided by the PearlDiver database, it is also not without additional inherent bias given the inter-rater variability and reliability concerns of these classifications. Our matching criteria instead were carefully selected to include the most relevant patient characteristics based on existing literature (i.e., age, gender, DM, obesity, tobacco use, and CKD) and a high- vs. low-energy fracture criterion to account for the differing fracture types found between IMF- and PF-treated patients in our study. The number of IMF cases also precludes meaningful sub-analysis of exclusively higher-risk cases (i.e., patients with comorbidities or specific fracture patterns). Finally, the very small percentage of percutaneous approaches among PF patients precludes the possibility of including operative approach (open vs. percutaneous PF) as a matching criteria, as there are insufficient percutaneously treated PF patients to match to percutaneously treated IMF patients with our specified matching process.

## 5. Conclusions

This study suggests that the use of fibular IMF is steadily rising. Moreover, we found that IMF and PF were associated with treating different injury patterns. IMF use was more often associated with treating higher energy injuries, those with soft tissue disruption, fibular injuries associated with tibial shaft fractures, and medically complex patients with comorbidities that may impair wound healing. Cost data suggests that fibular IMF appears to be a cheaper alternative to PF that yields comparable outcomes for treatment of distal fibular fractures; however, additional cost and efficacy studies are required.

## Figures and Tables

**Table 1:** Fracture types associated with fibular intramedullary fixation (IMF) versus plate fixation (PF) between October 2015 and April 2021.

	Fracture type	IMF (*N* = 2294)^1^	PF (*N* = 37035)^1^	*p* value
Fracture diagnoses more common among IMF patients	Open fracture (lower leg/ankle)	657 (28.6%)	5907 (15.9%)	**<0.001**
	Tibia shaft	1547 (67.4%)	5418 (14.6%)	**<0.001**
	Fibula shaft	1487 (64.8%)	8430 (22.8%)	**<0.001**
	Other upper and lower fibula	779 (34.0%)	7871 (21.3%)	**<0.001**
	Lower tibia	752 (32.8%)	4432 (12.0%)	**<0.001**
	Upper tibia	213 (9.3%)	1440 (3.9%)	**<0.001**
	Pilon	204 (8.9%)	2611 (7.1%)	**0.001**
	Lower fibula physeal	35 (1.5%)	393 (1.1%)	**0.035**
	Lower tibia physeal	30 (1.3%)	136 (0.4%)	**<0.001**
	Tibia stress/pathological	20 (0.9%)	85 (0.2%)	**<0.001**
	Other lower leg stress/pathologic	17 (0.7%)	125 (0.3%)	**0.002**
	Fibula stress/pathologic	16 (0.7%)	96 (0.3%)	**<0.001**
	Upper fibula physeal	14 (0.6%)	44 (0.1%)	**<0.001**

Fracture diagnoses more common among PF patients	Other/unspecified lower leg	354 (15.4%)	10179 (27.5%)	**<0.001**
	Bimalleolar	238 (10.4%)	14173 (38.3%)	**<0.001**
	Trimalleolar	223 (9.7%)	14233 (38.4%)	**<0.001**
	Lateral malleolus	184 (8.0%)	5435 (14.7%)	**<0.001**
	Medial malleolus	143 (6.2%)	4079 (11.0%)	**<0.001**
	Ankle stress/pathological	14 (0.6%)	566 (1.5%)	**<0.001**

No difference	Maisonneuve	14 (0.6%)	304 (0.8%)	0.276

^1^ *n* (%). Diagnoses identified and defined using International Classification of Diseases 10th revision (ICD-10) codes and descriptions. Bold *p* values are those considered statistically significant (i.e. *p* < 0.05).

**Table 2:** Demographics (nonmatched) of patients with distal fibula fractures undergoing surgical fixation.

Characteristic	IMF (*N* = 627)^1^	PF (*N* = 29703)^1^	*p* value
Age mean ± SD	59.4 ± 17.5	62.0 ± 15.9	**<0.001**
Age median (IQR)	63.0 (48.74)	65.0 (53.75)	
Age			
18–19	8 (1.3%)	163 (0.5%)	**0.021**
20–29	45 (7.2%)	1328 (4.5%)	**0.001**
30–39	55 (8.8%)	1957 (6.6%)	**0.030**
40–49	61 (9.7%)	2650 (8.9%)	0.483
50–59	102 (16.3%)	4974 (16.7%)	0.751
60–69	138 (22.0%)	6920 (23.3%)	0.450
70+	218 (34.8%)	11711 (39.4%)	**0.018**
Female	407 (64.9%)	20699 (69.7%)	**0.010**
ECI score	7.11 ± 4.77	6.62 ± 4.34	**0.005**
Diabetes	287 (45.8%)	13171 (44.3%)	0.475
Diabetes (complicated)	175 (27.9%)	7465 (25.1%)	0.113
Tobacco use	220 (35.1%)	9711 (32.7%)	0.206
Obesity	181 (28.9%)	7969 (26.8%)	0.254
Chronic kidney disease	152 (24.2%)	5961 (20.1%)	**0.010**
Osteoporosis	89 (14.2%)	3621 (12.2%)	0.130
Surgical approach			
Open	446 (71.1%)	29221 (98.4%)	**<0.001**
Percutaneous	181 (28.9%)	482 (1.6%)	**<0.001**
Region			
West	107 (17.1%)	5920 (19.9%)	0.075
Midwest	110 (17.5%)	6078 (20.5%)	0.073
South	298 (47.5%)	12420 (41.8%)	**0.004**
Northeast	108 (17.2%)	5116 (17.2%)	0.999
Unknown	4 (0.6%)	169 (0.6%)	0.820

^1^ *n* (%). IMF: intramedullary fixation. PF: plate fixation. ECI: Elixhauser Comorbidity Index. SD: standard deviation. IQR: interquartile range. Bold *p* values are those considered statistically significant (i.e. *p* < 0.05).

**Table 3:** Rates of postoperative complications, time to surgery, and cost by fixation method.

Event	IMF (*N* = 316)^1^	PF (*N* = 1254)^1^	*p* value
Infection	28 (8.9%)	87 (6.9%)	0.241
Nonunion	10 (3.2%)	25 (2.0%)	0.208
Malunion	4 (1.3%)	5 (0.4%)	0.068
Revision	12 (3.8%)	26 (2.1%)	0.075
Hardware removal	25 (7.9%)	91 (7.3%)	0.691
Pulmonary embolism	1 (0.3%)	1 (0.1%)	0.292
Deep vein thrombosis	4 (1.3%)	18 (1.4%)	0.819
Mean days to surgery	3.17 ± 4.68	3.24 ± 11.55	0.922
90-day cost (in USD)			
Mean ± SD	$6,585.58 ± $10,908.50	$9,217.24 ± $17,772.32	**0.012**
Median (IQR)	$3,017.00 ($925.00, $7,348.00)	$3,156.00 ($1,014.00, $9,663.00)	—

^1^ *n* (%). IMF: intramedullary fixation. PF: plate fixation. SD: standard deviation. IQR: interquartile range. Bold *p*-values are those considered statistically significant (i.e. *p* < 0.05).

## Data Availability

The administrative claims data supporting the results of this study were obtained from the PearlDiver Mariner Database via the PearlDiver software, which is publicly available with subscription at https://pearldiverinc.com.

## Supplementary Materials

Supplementary Materials A comprehensive list of the International Classification of Diseases, 10th Revision, Clinical Modification and Procedural Coding System (ICD-10-CM and ICD-10-PCS) codes and Current Procedural Terminology (CPT) codes billing codes used to query the PearlDiver database in this study can be found in Supplemental Tables 1–4 in the provided Supplementary Materials. A summary of the patient demographics of the matched IMF and PF groups used for the matched analysis along with the associated chi-squared test results demonstrating no significant differences between matched groups with respect to any matching criteria can be found in Supplemental Table 5 in the provided Supplementary Materials.
