# Supplementary material for: Current Trends in the Surgical Treatment of Fibular Fractures: A National Database Study of Intramedullary vs. Plate Fixation Practice Patterns, Complications, and Cost
Source: Adv Orthop. 2024 Jul 13;2024:7506557. doi: 10.1155/2024/7506557 (PMC11260212; doi:10.1155/2024/7506557)
Supplement: Supplementary Materials — A comprehensive list of the International Classification of Diseases, 10th Revision, Clinical Modification and Procedural Coding System (ICD-10-CM and ICD-10-PCS) codes and Current Procedural Terminology (CPT) codes billing codes used to query the PearlDiver database in this study can be found in Supplemental Tables 1–4 in the provided Supplementary Materials. A summary of the patient demographics of the matched IMF and PF groups used for the matched analysis along with the associated chi-squared test results demonstrating no significant differences between matched groups with respect to any matching criteria can be found in Supplemental Table 5 in the provided Supplementary Materials. [file 7506557.f1.docx]

| **Supplemental Table 1.** ICD-10-PCS Codes Corresponding to Plate and Intramedullary Fixation of the Fibula | |
| --- | --- |
| **Code** | **Code Description** |
| 0QHJ04Z | Insertion of Internal Fixation Device into Right Fibula Open Approach |
| 0QHK04Z | Insertion of Internal Fixation Device into Left Fibula Open Approach |
| 0QHJ06Z | Insertion of Intramedullary Internal Fixation Device into Right Fibula Open Approach |
| 0QHK06Z | Insertion of Intramedullary Internal Fixation Device into Left Fibula Open Approach |
| 0QHJ34Z | Insertion of Internal Fixation Device into Right Fibula, Percutaneous Approach |
| 0QHK34Z | Insertion of Internal Fixation Device into Right Fibula, Percutaneous Approach |
| 0QHJ36Z | Insertion of Intramedullary Internal Fixation Device into Right Fibula, Percutaneous Approach |
| 0QHK36Z | Insertion of Intramedullary Internal Fixation Device into Left Fibula, Percutaneous Approach |
| 0QHJ44Z | Insertion of Internal Fixation Device into Right Fibula, Endoscopic Percutaneous approach |
| 0QHK44Z | Insertion of Internal Fixation Device into Left Fibula, Endoscopic Percutaneous approach |
| 0QHJ46Z | Insertion of Intramedullary Internal Fixation Device into Right Fibula, Endoscopic Percutaneous approach |
| 0QHK46Z | Insertion of Intramedullary Internal Fixation Device into Left Fibula, Endoscopic Percutaneous approach |
| 0QSJ04Z | Reposition Right Fibula with Internal Fixation Device, Open Approach |
| 0QSK04Z | Reposition Left Fibula with Internal Fixation Device , Open Approach |
| 0QSJ06Z | Reposition Right Fibula with Intramedullary Internal Fixation Device, Open Approach |
| 0QSK6Z | Reposition Left Fibula with Intramedullary Internal Fixation Device, Open Approach |
| 0QSJ34Z | Reposition Right Fibula with Internal Fixation Device, Percutaneous Approach |
| 0QSK34Z | Reposition Left Fibula with Internal Fixation Device, Percutaneous Approach |
| 0QSJ36Z | Reposition Right Fibula with Intramedullary Internal Fixation Device, Percutaneous Approach |
| 0QSK36Z | Reposition Left Fibula with Intramedullary Internal Fixation Device, Percutaneous Approach |
| 0QSJ44Z | Reposition Right Fibula with Internal Fixation Device, Endoscopic Percutaneous Approach |
| 0QSK44Z | Reposition Left Fibula with Internal Fixation Device, Endoscopic Percutaneous Approach |
| 0QSJ46Z | Reposition Right Fibula with Intramedullary Internal Fixation Device, Endoscopic Percutaneous Approach |
| 0QSK46Z | Reposition Left Fibula with Intramedullary Internal Fixation Device, Endoscopic Percutaneous Approach |

| **Supplemental Table 2: Lower Leg and Ankle Fracture Diagnoses Diagnosis Codes** | |
| --- | --- |
| Fracture Description | Diagnosis Codes |
| Open Fracture (Lower Leg/Ankle) | ICD-10-D-S82101B, ICD-10-D-S82101C, ICD-10-D-S82102B, ICD-10-D-S82102C, ICD-10-D-S82109B, ICD-10-D-S82109C, ICD-10-D-S82111B, ICD-10-D-S82111C, ICD-10-D-S82112B, ICD-10-D-S82112C, ICD-10-D-S82113B, ICD-10-D-S82113C, ICD-10-D-S82114B, ICD-10-D-S82114C, ICD-10-D-S82115B, ICD-10-D-S82115C, ICD-10-D-S82116B, ICD-10-D-S82116C, ICD-10-D-S82121B, ICD-10-D-S82121C, ICD-10-D-S82122B, ICD-10-D-S82122C, ICD-10-D-S82123B, ICD-10-D-S82123C, ICD-10-D-S82124B, ICD-10-D-S82124C, ICD-10-D-S82125B, ICD-10-D-S82125C, ICD-10-D-S82126B, ICD-10-D-S82126C, ICD-10-D-S82131B, ICD-10-D-S82131C, ICD-10-D-S82132B, ICD-10-D-S82132C, ICD-10-D-S82133B, ICD-10-D-S82133C, ICD-10-D-S82134B, ICD-10-D-S82134C, ICD-10-D-S82135B, ICD-10-D-S82135C, ICD-10-D-S82136B, ICD-10-D-S82141B, ICD-10-D-S82141C, ICD-10-D-S82142B, ICD-10-D-S82142C, ICD-10-D-S82143B, ICD-10-D-S82143C, ICD-10-D-S82144B, ICD-10-D-S82144C, ICD-10-D-S82145B, ICD-10-D-S82145C, ICD-10-D-S82146B, ICD-10-D-S82151B, ICD-10-D-S82151C, ICD-10-D-S82152B, ICD-10-D-S82152C, ICD-10-D-S82153B, ICD-10-D-S82153C, ICD-10-D-S82154B, ICD-10-D-S82154C, ICD-10-D-S82155B, ICD-10-D-S82155C, ICD-10-D-S82156B, ICD-10-D-S82156C, ICD-10-D-S82191B, ICD-10-D-S82191C, ICD-10-D-S82192B, ICD-10-D-S82192C, ICD-10-D-S82199B, ICD-10-D-S82199C, ICD-10-D-S82201B, ICD-10-D-S82201C, ICD-10-D-S82202B, ICD-10-D-S82202C, ICD-10-D-S82209B, ICD-10-D-S82209C, ICD-10-D-S82221B, ICD-10-D-S82221C, ICD-10-D-S82222B, ICD-10-D-S82222C, ICD-10-D-S82223B, ICD-10-D-S82223C, ICD-10-D-S82224B, ICD-10-D-S82224C, ICD-10-D-S82225B, ICD-10-D-S82225C, ICD-10-D-S82226B, ICD-10-D-S82231B, ICD-10-D-S82231C, ICD-10-D-S82232B, ICD-10-D-S82232C, ICD-10-D-S82233B, ICD-10-D-S82233C, ICD-10-D-S82234B, ICD-10-D-S82234C, ICD-10-D-S82235B, ICD-10-D-S82235C, ICD-10-D-S82236B, ICD-10-D-S82236C, ICD-10-D-S82241B, ICD-10-D-S82241C, ICD-10-D-S82242B, ICD-10-D-S82242C, ICD-10-D-S82243B, ICD-10-D-S82243C, ICD-10-D-S82244B, ICD-10-D-S82244C, ICD-10-D-S82245B, ICD-10-D-S82245C, ICD-10-D-S82246B, ICD-10-D-S82251B, ICD-10-D-S82251C, ICD-10-D-S82252B, ICD-10-D-S82252C, ICD-10-D-S82253B, ICD-10-D-S82253C, ICD-10-D-S82254B, ICD-10-D-S82254C, ICD-10-D-S82255B, ICD-10-D-S82255C, ICD-10-D-S82256B, ICD-10-D-S82256C, ICD-10-D-S82261B, ICD-10-D-S82261C, ICD-10-D-S82262B, ICD-10-D-S82262C, ICD-10-D-S82263B, ICD-10-D-S82263C, ICD-10-D-S82264B, ICD-10-D-S82264C, ICD-10-D-S82265B, ICD-10-D-S82265C, ICD-10-D-S82291B, ICD-10-D-S82291C, ICD-10-D-S82292B, ICD-10-D-S82292C, ICD-10-D-S82299B, ICD-10-D-S82299C, ICD-10-D-S82301B, ICD-10-D-S82301C, ICD-10-D-S82302B, ICD-10-D-S82302C, ICD-10-D-S82309B, ICD-10-D-S82309C, ICD-10-D-S82391B, ICD-10-D-S82391C, ICD-10-D-S82392B, ICD-10-D-S82392C, ICD-10-D-S82399B, ICD-10-D-S82399C, ICD-10-D-S82401B, ICD-10-D-S82401C, ICD-10-D-S82402B, ICD-10-D-S82402C, ICD-10-D-S82409B, ICD-10-D-S82409C, ICD-10-D-S82421B, ICD-10-D-S82421C, ICD-10-D-S82422B, ICD-10-D-S82422C, ICD-10-D-S82423B, ICD-10-D-S82423C, ICD-10-D-S82424B, ICD-10-D-S82424C, ICD-10-D-S82425B, ICD-10-D-S82425C, ICD-10-D-S82431B, ICD-10-D-S82431C, ICD-10-D-S82432B, ICD-10-D-S82432C, ICD-10-D-S82433B, ICD-10-D-S82433C, ICD-10-D-S82434B, ICD-10-D-S82434C, ICD-10-D-S82435B, ICD-10-D-S82435C, ICD-10-D-S82436B, ICD-10-D-S82436C, ICD-10-D-S82441B, ICD-10-D-S82441C, ICD-10-D-S82442B, ICD-10-D-S82442C, ICD-10-D-S82443B, ICD-10-D-S82444B, ICD-10-D-S82444C, ICD-10-D-S82445B, ICD-10-D-S82445C, ICD-10-D-S82446B, ICD-10-D-S82451B, ICD-10-D-S82451C, ICD-10-D-S82452B, ICD-10-D-S82452C, ICD-10-D-S82453B, ICD-10-D-S82453C, ICD-10-D-S82454B, ICD-10-D-S82454C, ICD-10-D-S82455B, ICD-10-D-S82455C, ICD-10-D-S82456B, ICD-10-D-S82456C, ICD-10-D-S82461B, ICD-10-D-S82461C, ICD-10-D-S82462B, ICD-10-D-S82462C, ICD-10-D-S82463B, ICD-10-D-S82464B, ICD-10-D-S82464C, ICD-10-D-S82465B, ICD-10-D-S82465C, ICD-10-D-S82466C, ICD-10-D-S82491B, ICD-10-D-S82491C, ICD-10-D-S82492B, ICD-10-D-S82492C, ICD-10-D-S82499B, ICD-10-D-S82499C, ICD-10-D-S8251XB, ICD-10-D-S8251XC, ICD-10-D-S8252XB, ICD-10-D-S8252XC, ICD-10-D-S8253XB, ICD-10-D-S8253XC, ICD-10-D-S8254XB, ICD-10-D-S8254XC, ICD-10-D-S8255XB, ICD-10-D-S8255XC, ICD-10-D-S8256XB, ICD-10-D-S8256XC, ICD-10-D-S8261XB, ICD-10-D-S8261XC, ICD-10-D-S8262XB, ICD-10-D-S8262XC, ICD-10-D-S8263XB, ICD-10-D-S8263XC, ICD-10-D-S8264XB, ICD-10-D-S8264XC, ICD-10-D-S8265XB, ICD-10-D-S8265XC, ICD-10-D-S8266XB, ICD-10-D-S8266XC, ICD-10-D-S82831B, ICD-10-D-S82831C, ICD-10-D-S82832B, ICD-10-D-S82832C, ICD-10-D-S82839B, ICD-10-D-S82839C, ICD-10-D-S82841B, ICD-10-D-S82841C, ICD-10-D-S82842B, ICD-10-D-S82842C, ICD-10-D-S82843B, ICD-10-D-S82843C, ICD-10-D-S82844B, ICD-10-D-S82844C, ICD-10-D-S82845B, ICD-10-D-S82845C, ICD-10-D-S82846B, ICD-10-D-S82846C, ICD-10-D-S82851B, ICD-10-D-S82851C, ICD-10-D-S82852B, ICD-10-D-S82852C, ICD-10-D-S82853B, ICD-10-D-S82853C, ICD-10-D-S82854B, ICD-10-D-S82854C, ICD-10-D-S82855B, ICD-10-D-S82855C, ICD-10-D-S82856B, ICD-10-D-S82856C, ICD-10-D-S82861B, ICD-10-D-S82861C, ICD-10-D-S82862B, ICD-10-D-S82862C, ICD-10-D-S82864B, ICD-10-D-S82864C, ICD-10-D-S82865B, ICD-10-D-S82865C, ICD-10-D-S82866C, ICD-10-D-S82871B, ICD-10-D-S82871C, ICD-10-D-S82872B, ICD-10-D-S82872C, ICD-10-D-S82873B, ICD-10-D-S82873C, ICD-10-D-S82874B, ICD-10-D-S82874C, ICD-10-D-S82875B, ICD-10-D-S82875C, ICD-10-D-S82876B, ICD-10-D-S82891B, ICD-10-D-S82891C, ICD-10-D-S82892B, ICD-10-D-S82892C, ICD-10-D-S82899B, ICD-10-D-S82899C, ICD-10-D-S8290XB, ICD-10-D-S8290XC, ICD-10-D-S8291XB, ICD-10-D-S8291XC, ICD-10-D-S8292XB, ICD-10-D-S8292XC, ICD-10-D-S92101B, ICD-10-D-S92102B, ICD-10-D-S92109B, ICD-10-D-S92111B, ICD-10-D-S92112B, ICD-10-D-S92113B, ICD-10-D-S92114B, ICD-10-D-S92115B, ICD-10-D-S92121B, ICD-10-D-S92122B, ICD-10-D-S92123B, ICD-10-D-S92124B, ICD-10-D-S92125B, ICD-10-D-S92126B, ICD-10-D-S92131B, ICD-10-D-S92132B, ICD-10-D-S92133B, ICD-10-D-S92134B, ICD-10-D-S92135B, ICD-10-D-S92141B, ICD-10-D-S92142B, ICD-10-D-S92143B, ICD-10-D-S92144B, ICD-10-D-S92145B, ICD-10-D-S92146B, ICD-10-D-S92151B, ICD-10-D-S92152B, ICD-10-D-S92153B, ICD-10-D-S92154B, ICD-10-D-S92155B, ICD-10-D-S92156B, ICD-10-D-S92191B, ICD-10-D-S92192B, ICD-10-D-S92199B |
| Tibia Shaft | ICD-10-D-S82202A, ICD-10-D-S82202B, ICD-10-D-S82201A, ICD-10-D-S82201B, ICD-10-D-S82201C, ICD-10-D-S82202C, ICD-10-D-S82209A, ICD-10-D-S82209B, ICD-10-D-S82209C, ICD-10-D-S82221A, ICD-10-D-S82221B, ICD-10-D-S82221C, ICD-10-D-S82222A, ICD-10-D-S82222B, ICD-10-D-S82222C, ICD-10-D-S82223A, ICD-10-D-S82223B, ICD-10-D-S82223C, ICD-10-D-S82224A, ICD-10-D-S82224B, ICD-10-D-S82224C, ICD-10-D-S82225A, ICD-10-D-S82225B, ICD-10-D-S82225C, ICD-10-D-S82226A, ICD-10-D-S82226B, ICD-10-D-S82231A, ICD-10-D-S82231B, ICD-10-D-S82231C, ICD-10-D-S82232A, ICD-10-D-S82232B, ICD-10-D-S82232C, ICD-10-D-S82233A, ICD-10-D-S82233B, ICD-10-D-S82233C, ICD-10-D-S82234A, ICD-10-D-S82234B, ICD-10-D-S82234C, ICD-10-D-S82235A, ICD-10-D-S82235B, ICD-10-D-S82235C, ICD-10-D-S82236A, ICD-10-D-S82236B, ICD-10-D-S82236C, ICD-10-D-S82241A, ICD-10-D-S82241B, ICD-10-D-S82241C, ICD-10-D-S82242A, ICD-10-D-S82242B, ICD-10-D-S82242C, ICD-10-D-S82243A, ICD-10-D-S82243B, ICD-10-D-S82243C, ICD-10-D-S82244A, ICD-10-D-S82244B, ICD-10-D-S82244C, ICD-10-D-S82245A, ICD-10-D-S82245B, ICD-10-D-S82245C, ICD-10-D-S82246A, ICD-10-D-S82246B, ICD-10-D-S82251A, ICD-10-D-S82251B, ICD-10-D-S82251C, ICD-10-D-S82252A, ICD-10-D-S82252B, ICD-10-D-S82252C, ICD-10-D-S82253A, ICD-10-D-S82253B, ICD-10-D-S82253C, ICD-10-D-S82254A, ICD-10-D-S82254B, ICD-10-D-S82254C, ICD-10-D-S82255A, ICD-10-D-S82255B, ICD-10-D-S82255C, ICD-10-D-S82256A, ICD-10-D-S82256B, ICD-10-D-S82256C, ICD-10-D-S82261A, ICD-10-D-S82261B, ICD-10-D-S82261C, ICD-10-D-S82262A, ICD-10-D-S82262B, ICD-10-D-S82262C, ICD-10-D-S82263A, ICD-10-D-S82263B, ICD-10-D-S82263C, ICD-10-D-S82264A, ICD-10-D-S82264B, ICD-10-D-S82264C, ICD-10-D-S82265A, ICD-10-D-S82265B, ICD-10-D-S82265C, ICD-10-D-S82266A, ICD-10-D-S82291A, ICD-10-D-S82291B, ICD-10-D-S82291C, ICD-10-D-S82292A, ICD-10-D-S82292B, ICD-10-D-S82292C, ICD-10-D-S82299A, ICD-10-D-S82299B, ICD-10-D-S82299C |
| Fibula Shaft | ICD-10-D-S82401A, ICD-10-D-S82401B, ICD-10-D-S82401C, ICD-10-D-S82402A, ICD-10-D-S82402B, ICD-10-D-S82402C, ICD-10-D-S82409A, ICD-10-D-S82409B, ICD-10-D-S82409C, ICD-10-D-S82421A, ICD-10-D-S82421B, ICD-10-D-S82421C, ICD-10-D-S82422A, ICD-10-D-S82422B, ICD-10-D-S82422C, ICD-10-D-S82423A, ICD-10-D-S82423B, ICD-10-D-S82423C, ICD-10-D-S82424A, ICD-10-D-S82424B, ICD-10-D-S82424C, ICD-10-D-S82425A, ICD-10-D-S82425B, ICD-10-D-S82425C, ICD-10-D-S82426A, ICD-10-D-S82431A, ICD-10-D-S82431B, ICD-10-D-S82431C, ICD-10-D-S82432A, ICD-10-D-S82432B, ICD-10-D-S82432C, ICD-10-D-S82433A, ICD-10-D-S82433B, ICD-10-D-S82433C, ICD-10-D-S82434A, ICD-10-D-S82434B, ICD-10-D-S82434C, ICD-10-D-S82435A, ICD-10-D-S82435B, ICD-10-D-S82435C, ICD-10-D-S82436A, ICD-10-D-S82436B, ICD-10-D-S82436C, ICD-10-D-S82441A, ICD-10-D-S82441B, ICD-10-D-S82441C, ICD-10-D-S82442A, ICD-10-D-S82442B, ICD-10-D-S82442C, ICD-10-D-S82443A, ICD-10-D-S82443B, ICD-10-D-S82444A, ICD-10-D-S82444B, ICD-10-D-S82444C, ICD-10-D-S82445A, ICD-10-D-S82445B, ICD-10-D-S82445C, ICD-10-D-S82446A, ICD-10-D-S82446B, ICD-10-D-S82451A, ICD-10-D-S82451B, ICD-10-D-S82451C, ICD-10-D-S82452A, ICD-10-D-S82452B, ICD-10-D-S82452C, ICD-10-D-S82453A, ICD-10-D-S82453B, ICD-10-D-S82453C, ICD-10-D-S82454A, ICD-10-D-S82454B, ICD-10-D-S82454C, ICD-10-D-S82455A, ICD-10-D-S82455B, ICD-10-D-S82455C, ICD-10-D-S82456A, ICD-10-D-S82456B, ICD-10-D-S82456C, ICD-10-D-S82461A, ICD-10-D-S82461B, ICD-10-D-S82461C, ICD-10-D-S82462A, ICD-10-D-S82462B, ICD-10-D-S82462C, ICD-10-D-S82463A, ICD-10-D-S82463B, ICD-10-D-S82464A, ICD-10-D-S82464B, ICD-10-D-S82464C, ICD-10-D-S82465A, ICD-10-D-S82465B, ICD-10-D-S82465C, ICD-10-D-S82466A, ICD-10-D-S82466C, ICD-10-D-S82491A, ICD-10-D-S82491B, ICD-10-D-S82491C, ICD-10-D-S82492A, ICD-10-D-S82492B, ICD-10-D-S82492C, ICD-10-D-S82499A, ICD-10-D-S82499B, ICD-10-D-S82499C |
| Other Upper and Lower Fibula | ICD-10-D-S82831A, ICD-10-D-S82831B, ICD-10-D-S82831C, ICD-10-D-S82832A, ICD-10-D-S82832B, ICD-10-D-S82832C, ICD-10-D-S82839A, ICD-10-D-S82839B, ICD-10-D-S82839C |
| Lower Tibia | ICD-10-D-S82302A, ICD-10-D-S82301A, ICD-10-D-S82301B, ICD-10-D-S82301C, ICD-10-D-S82302B, ICD-10-D-S82302C, ICD-10-D-S82309A, ICD-10-D-S82309B, ICD-10-D-S82309C, ICD-10-D-S82311A, ICD-10-D-S82312A, ICD-10-D-S82319A, ICD-10-D-S82391A, ICD-10-D-S82391B, ICD-10-D-S82391C, ICD-10-D-S82392A, ICD-10-D-S82392B, ICD-10-D-S82392C, ICD-10-D-S82399A, ICD-10-D-S82399B, ICD-10-D-S82399C |
| Upper Tibia | ICD-10-D-S82101A, ICD-10-D-S82101B, ICD-10-D-S82101C, ICD-10-D-S82102A, ICD-10-D-S82102B, ICD-10-D-S82102C, ICD-10-D-S82109A, ICD-10-D-S82109B, ICD-10-D-S82109C, ICD-10-D-S82111A, ICD-10-D-S82111B, ICD-10-D-S82111C, ICD-10-D-S82112A, ICD-10-D-S82112B, ICD-10-D-S82112C, ICD-10-D-S82113A, ICD-10-D-S82113B, ICD-10-D-S82113C, ICD-10-D-S82114A, ICD-10-D-S82114B, ICD-10-D-S82114C, ICD-10-D-S82115A, ICD-10-D-S82115B, ICD-10-D-S82115C, ICD-10-D-S82116A, ICD-10-D-S82116B, ICD-10-D-S82116C, ICD-10-D-S82121A, ICD-10-D-S82121B, ICD-10-D-S82121C, ICD-10-D-S82122A, ICD-10-D-S82122B, ICD-10-D-S82122C, ICD-10-D-S82123A, ICD-10-D-S82123B, ICD-10-D-S82123C, ICD-10-D-S82124A, ICD-10-D-S82124B, ICD-10-D-S82124C, ICD-10-D-S82125A, ICD-10-D-S82125B, ICD-10-D-S82125C, ICD-10-D-S82126A, ICD-10-D-S82126B, ICD-10-D-S82126C, ICD-10-D-S82131A, ICD-10-D-S82131B, ICD-10-D-S82131C, ICD-10-D-S82132A, ICD-10-D-S82132B, ICD-10-D-S82132C, ICD-10-D-S82133A, ICD-10-D-S82133B, ICD-10-D-S82133C, ICD-10-D-S82134A, ICD-10-D-S82134B, ICD-10-D-S82134C, ICD-10-D-S82135A, ICD-10-D-S82135B, ICD-10-D-S82135C, ICD-10-D-S82136A, ICD-10-D-S82136B, ICD-10-D-S82141A, ICD-10-D-S82141B, ICD-10-D-S82141C, ICD-10-D-S82142A, ICD-10-D-S82142B, ICD-10-D-S82142C, ICD-10-D-S82143A, ICD-10-D-S82143B, ICD-10-D-S82143C, ICD-10-D-S82144A, ICD-10-D-S82144B, ICD-10-D-S82144C, ICD-10-D-S82145A, ICD-10-D-S82145B, ICD-10-D-S82145C, ICD-10-D-S82146A, ICD-10-D-S82146B, ICD-10-D-S82151A, ICD-10-D-S82151B, ICD-10-D-S82151C, ICD-10-D-S82152A, ICD-10-D-S82152B, ICD-10-D-S82152C, ICD-10-D-S82153A, ICD-10-D-S82153B, ICD-10-D-S82153C, ICD-10-D-S82154A, ICD-10-D-S82154B, ICD-10-D-S82154C, ICD-10-D-S82155A, ICD-10-D-S82155B, ICD-10-D-S82155C, ICD-10-D-S82156A, ICD-10-D-S82156B, ICD-10-D-S82156C, ICD-10-D-S82161A, ICD-10-D-S82162A, ICD-10-D-S82169A, ICD-10-D-S82191A, ICD-10-D-S82191B, ICD-10-D-S82191C, ICD-10-D-S82192A, ICD-10-D-S82192B, ICD-10-D-S82192C, ICD-10-D-S82199A, ICD-10-D-S82199B, ICD-10-D-S82199C |
| Pilon | ICD-10-D-S82871A, ICD-10-D-S82871B, ICD-10-D-S82871C, ICD-10-D-S82872A, ICD-10-D-S82872B, ICD-10-D-S82872C, ICD-10-D-S82873A, ICD-10-D-S82873B, ICD-10-D-S82873C, ICD-10-D-S82874A, ICD-10-D-S82874B, ICD-10-D-S82874C, ICD-10-D-S82875A, ICD-10-D-S82875B, ICD-10-D-S82875C, ICD-10-D-S82876A, ICD-10-D-S82876B |
| Lower Fibula Physeal | ICD-10-D-S89201A, ICD-10-D-S89202A, ICD-10-D-S89209A, ICD-10-D-S89211A, ICD-10-D-S89212A, ICD-10-D-S89219A, ICD-10-D-S89221A, ICD-10-D-S89222A, ICD-10-D-S89229A, ICD-10-D-S89291A, ICD-10-D-S89292A, ICD-10-D-S89299A |
| Lower Tibia Physeal | ICD-10-D-S89101A, ICD-10-D-S89102A, ICD-10-D-S89109A, ICD-10-D-S89111A, ICD-10-D-S89112A, ICD-10-D-S89119A, ICD-10-D-S89121A, ICD-10-D-S89122A, ICD-10-D-S89129A, ICD-10-D-S89131A, ICD-10-D-S89132A, ICD-10-D-S89139A, ICD-10-D-S89141A, ICD-10-D-S89142A, ICD-10-D-S89149A, ICD-10-D-S89191A, ICD-10-D-S89192A, ICD-10-D-S89199A |
| Tibia Stress/Pathological | ICD-10-D-M84361A, ICD-10-D-M84362A, ICD-10-D-M84369A, ICD-10-D-M84461A, ICD-10-D-M84462A, ICD-10-D-M84469A, ICD-10-D-M84561A, ICD-10-D-M84562A, ICD-10-D-M84569A, ICD-10-D-M84661A, ICD-10-D-M84662A, ICD-10-D-M84669A |
| Other Lower Leg Stress/Pathologic | ICD-10-D-M80061A, ICD-10-D-M80062A, ICD-10-D-M80069A, ICD-10-D-M80861A, ICD-10-D-M80862A, ICD-10-D-M80869A |
| Fibula Stress/Pathologic | ICD-10-D-M84363A, ICD-10-D-M84364A, ICD-10-D-M84369A, ICD-10-D-M84463A, ICD-10-D-M84464A, ICD-10-D-M84469A, ICD-10-D-M84563A, ICD-10-D-M84564A, ICD-10-D-M84569A, ICD-10-D-M84663A, ICD-10-D-M84664A, ICD-10-D-M84669A |
| Upper Fibula Physeal | ICD-10-D-S89201A, ICD-10-D-S89202A, ICD-10-D-S89209A, ICD-10-D-S89211A, ICD-10-D-S89212A, ICD-10-D-S89219A, ICD-10-D-S89221A, ICD-10-D-S89222A, ICD-10-D-S89229A, ICD-10-D-S89291A, ICD-10-D-S89292A, ICD-10-D-S89299A |
| Other/Unspecified Lower Leg | ICD-10-D-S82891A, ICD-10-D-S82891B, ICD-10-D-S82891C, ICD-10-D-S82892A, ICD-10-D-S82892B, ICD-10-D-S82892C, ICD-10-D-S82899A, ICD-10-D-S82899B, ICD-10-D-S82899C, ICD-10-D-S8290XA, ICD-10-D-S8290XB, ICD-10-D-S8290XC, ICD-10-D-S8291XA, ICD-10-D-S8291XB, ICD-10-D-S8291XC, ICD-10-D-S8292XA, ICD-10-D-S8292XB, ICD-10-D-S8292XC |
| Bimalleolar | ICD-10-D-S82841A, ICD-10-D-S82841B, ICD-10-D-S82841C, ICD-10-D-S82842A, ICD-10-D-S82842B, ICD-10-D-S82842C, ICD-10-D-S82843A, ICD-10-D-S82843B, ICD-10-D-S82843C, ICD-10-D-S82844A, ICD-10-D-S82844B, ICD-10-D-S82844C, ICD-10-D-S82845A, ICD-10-D-S82845B, ICD-10-D-S82845C, ICD-10-D-S82846A, ICD-10-D-S82846B, ICD-10-D-S82846C |
| Trimalleolar | ICD-10-D-S82851A, ICD-10-D-S82851B, ICD-10-D-S82851C, ICD-10-D-S82852A, ICD-10-D-S82852B, ICD-10-D-S82852C, ICD-10-D-S82853A, ICD-10-D-S82853B, ICD-10-D-S82853C, ICD-10-D-S82854A, ICD-10-D-S82854B, ICD-10-D-S82854C, ICD-10-D-S82855A, ICD-10-D-S82855B, ICD-10-D-S82855C, ICD-10-D-S82856A, ICD-10-D-S82856B, ICD-10-D-S82856C |
| Lateral Malleolus | ICD-10-D-S8261XA, ICD-10-D-S8261XB, ICD-10-D-S8261XC, ICD-10-D-S8262XA, ICD-10-D-S8262XB, ICD-10-D-S8262XC, ICD-10-D-S8265XB, ICD-10-D-S8263XA, ICD-10-D-S8263XB, ICD-10-D-S8263XC, ICD-10-D-S8264XA, ICD-10-D-S8264XB, ICD-10-D-S8264XC, ICD-10-D-S8265XA, ICD-10-D-S8265XC, ICD-10-D-S8266XA, ICD-10-D-S8266XB, ICD-10-D-S8266XC |
| Medial Malleolus | ICD-10-D-S8251XA, ICD-10-D-S8251XB, ICD-10-D-S8251XC, ICD-10-D-S8252XA, ICD-10-D-S8252XB, ICD-10-D-S8252XC, ICD-10-D-S8253XA, ICD-10-D-S8253XB, ICD-10-D-S8253XC, ICD-10-D-S8254XA, ICD-10-D-S8254XB, ICD-10-D-S8254XC, ICD-10-D-S8255XA, ICD-10-D-S8255XB, ICD-10-D-S8255XC, ICD-10-D-S8256XA, ICD-10-D-S8256XB, ICD-10-D-S8256XC |
| Ankle Stress/Pathological | ICD-10-D-M80071A, ICD-10-D-M80072A, ICD-10-D-M80079A, ICD-10-D-M80871A, ICD-10-D-M80872A, ICD-10-D-M80879A, ICD-10-D-M84371A, ICD-10-D-M84372A, ICD-10-D-M84373A, ICD-10-D-M84471A, ICD-10-D-M84472A, ICD-10-D-M84473A, ICD-10-D-M84571A, ICD-10-D-M84572A, ICD-10-D-M84573A, ICD-10-D-M84671A, ICD-10-D-M84672A, ICD-10-D-M84673A |
| Maisonneuve | ICD-10-D-S82862A, ICD-10-D-S82862C, ICD-10-D-S82862B, ICD-10-D-S82861A, ICD-10-D-S82861C, ICD-10-D-S82861B, ICD-10-D-S82863A, ICD-10-D-S82865A, ICD-10-D-S82865C, ICD-10-D-S82865B, ICD-10-D-S82864A, ICD-10-D-S82864C, ICD-10-D-S82864B, ICD-10-D-S82866A, ICD-10-D-S82866C |

| **Supplemental Table 3: Diagnosis Codes Used to Define Comorbidities** | |
| --- | --- |
| Comorbidity | Billing Codes (ICD-10-CM) |
| Diabetes | ICD-9-D-24900:ICD-9-D-25099, ICD-9-D-7902, ICD-9-D-79021, ICD-9-D-79022, ICD-9-D-79029, ICD-9-D-7915, ICD-9-D-7916, ICD-10-D-E080:ICD-10-D-E139 |
| Tobacco Use | ICD-9-D-3051, ICD-9-D-98984, ICD-9-D-V1582, ICD-9-D-3051, ICD-10-D-F17220, ICD-10-D-F17221, ICD-10-D-F17223, ICD-10-D-F17228, ICD-10-D-F17229, ICD-10-D-F17290, ICD-10-D-F17291, ICD-10-D-F17293, ICD-10-D-F17298, ICD-10-D-F17299, ICD-10-D-Z720, ICD-10-D-F17200, ICD-10-D-F17201, ICD-10-D-F17203, ICD-10-D-F17208, ICD-10-D-F17209, ICD-10-D-F17210, ICD-10-D-F17211, ICD-10-D-F17213, ICD-10-D-F17218, ICD-10-D-F17219, ICD-10-D-F17220, ICD-10-D-F17221, ICD-10-D-F17223, ICD-10-D-F17228, ICD-10-D-F17229, ICD-10-D-F17290, ICD-10-D-F17291, ICD-10-D-F17293, ICD-10-D-F17298, ICD-10-D-F17299, ICD-10-D-Z716, ICD-10-D-Z720, ICD-10-D-Z87891 |
| Obesity | ICD-9-D-2780, ICD-9-D-27800, ICD-9-D-27801, ICD-9-D-27802, ICD-9-D-27803, ICD-10-D-E660:ICD-10-D-E669 |
| Chronic Kidney Disease | ICD-9-D-585, ICD-9-D-5851, ICD-9-D-5852, ICD-9-D-5853, ICD-9-D-5854, ICD-9-D-5855, ICD-9-D-5856, ICD-9-D-5859, ICD-9-D-7925, ICD-10-D-N18:ICD-10-D-N189 |
| Osteoporosis | ICD-9-D-73300, ICD-9-D-73301, ICD-9-D-73302, ICD-9-D-73303, ICD-9-D-73309, ICD-10-D-Z87310, ICD-10-D-M818, ICD-10-D-M816, ICD-10-D-M810, ICD-10-D-M808AXS, ICD-10-D-M808AXP, ICD-10-D-M808AXK, ICD-10-D-M808AXG, ICD-10-D-M808AXD, ICD-10-D-M808AXA, ICD-10-D-M8088XS, ICD-10-D-M8088XP, ICD-10-D-M8088XK, ICD-10-D-M8088XG, ICD-10-D-M8088XD, ICD-10-D-M8088XA, ICD-10-D-M80879S, ICD-10-D-M80879P, ICD-10-D-M80879K, ICD-10-D-M80879G, ICD-10-D-M80879D, ICD-10-D-M80879A, ICD-10-D-M80872S, ICD-10-D-M80872P, ICD-10-D-M80872K, ICD-10-D-M80872G, ICD-10-D-M80872D, ICD-10-D-M80872A, ICD-10-D-M80871S, ICD-10-D-M80871P, ICD-10-D-M80871K, ICD-10-D-M80871G, ICD-10-D-M80871D, ICD-10-D-M80871A, ICD-10-D-M80869S, ICD-10-D-M80869P, ICD-10-D-M80869K, ICD-10-D-M80869G, ICD-10-D-M80869D, ICD-10-D-M80869A, ICD-10-D-M80862S, ICD-10-D-M80862P, ICD-10-D-M80862K, ICD-10-D-M80862G, ICD-10-D-M80862D, ICD-10-D-M80862A, ICD-10-D-M80861S, ICD-10-D-M80861P, ICD-10-D-M80861K, ICD-10-D-M80861G, ICD-10-D-M80861D, ICD-10-D-M80861A, ICD-10-D-M80859S, ICD-10-D-M80859P, ICD-10-D-M80859K, ICD-10-D-M80859G, ICD-10-D-M80859D, ICD-10-D-M80859A, ICD-10-D-M80852S, ICD-10-D-M80852P, ICD-10-D-M80852K, ICD-10-D-M80852G, ICD-10-D-M80852D, ICD-10-D-M80852A, ICD-10-D-M80851S, ICD-10-D-M80851P, ICD-10-D-M80851K, ICD-10-D-M80851G, ICD-10-D-M80851D, ICD-10-D-M80851A, ICD-10-D-M80849S, ICD-10-D-M80849P, ICD-10-D-M80849K, ICD-10-D-M80849G, ICD-10-D-M80849D, ICD-10-D-M80849A, ICD-10-D-M80842S, ICD-10-D-M80842P, ICD-10-D-M80842K, ICD-10-D-M80842G, ICD-10-D-M80842D, ICD-10-D-M80842A, ICD-10-D-M80841S, ICD-10-D-M80841P, ICD-10-D-M80841K, ICD-10-D-M80841G, ICD-10-D-M80841D, ICD-10-D-M80841A, ICD-10-D-M80839S, ICD-10-D-M80839P, ICD-10-D-M80839K, ICD-10-D-M80839G, ICD-10-D-M80839D, ICD-10-D-M80839A, ICD-10-D-M80832S, ICD-10-D-M80832P, ICD-10-D-M80832K, ICD-10-D-M80832G, ICD-10-D-M80832D, ICD-10-D-M80832A, ICD-10-D-M80831S, ICD-10-D-M80831P, ICD-10-D-M80831K, ICD-10-D-M80831G, ICD-10-D-M80831D, ICD-10-D-M80831A, ICD-10-D-M80829S, ICD-10-D-M80829P, ICD-10-D-M80829K, ICD-10-D-M80829G, ICD-10-D-M80829D, ICD-10-D-M80829A, ICD-10-D-M80822S, ICD-10-D-M80822P, ICD-10-D-M80822K, ICD-10-D-M80822G, ICD-10-D-M80822D, ICD-10-D-M80822A, ICD-10-D-M80821S, ICD-10-D-M80821P, ICD-10-D-M80821K, ICD-10-D-M80821G, ICD-10-D-M80821D, ICD-10-D-M80821A, ICD-10-D-M80819S, ICD-10-D-M80819P, ICD-10-D-M80819G, ICD-10-D-M80819D, ICD-10-D-M80819A, ICD-10-D-M80812S, ICD-10-D-M80812P, ICD-10-D-M80812K, ICD-10-D-M80812G, ICD-10-D-M80812D, ICD-10-D-M80812A, ICD-10-D-M80811S, ICD-10-D-M80811P, ICD-10-D-M80811K, ICD-10-D-M80811G, ICD-10-D-M80811D, ICD-10-D-M80811A, ICD-10-D-M8080XS, ICD-10-D-M8080XP, ICD-10-D-M8080XK, ICD-10-D-M8080XG, ICD-10-D-M8080XD, ICD-10-D-M8080XA, ICD-10-D-M800AXS, ICD-10-D-M800AXK, ICD-10-D-M800AXG, ICD-10-D-M800AXD, ICD-10-D-M800AXA, ICD-10-D-M8008XS, ICD-10-D-M8008XP, ICD-10-D-M8008XK, ICD-10-D-M8008XG, ICD-10-D-M8008XD, ICD-10-D-M8008XA, ICD-10-D-M80079S, ICD-10-D-M80079P, ICD-10-D-M80079K, ICD-10-D-M80079G, ICD-10-D-M80079D, ICD-10-D-M80079A, ICD-10-D-M80072S, ICD-10-D-M80072P, ICD-10-D-M80072K, ICD-10-D-M80072G, ICD-10-D-M80072D, ICD-10-D-M80072A, ICD-10-D-M80071S, ICD-10-D-M80071P, ICD-10-D-M80071K, ICD-10-D-M80071G, ICD-10-D-M80071D, ICD-10-D-M80071A, ICD-10-D-M80069S, ICD-10-D-M80069P, ICD-10-D-M80069K, ICD-10-D-M80069G, ICD-10-D-M80069D, ICD-10-D-M80069A, ICD-10-D-M80062S, ICD-10-D-M80062P, ICD-10-D-M80062K, ICD-10-D-M80062G, ICD-10-D-M80062D, ICD-10-D-M80062A, ICD-10-D-M80061S, ICD-10-D-M80061P, ICD-10-D-M80061K, ICD-10-D-M80061G, ICD-10-D-M80061D, ICD-10-D-M80061A, ICD-10-D-M80059S, ICD-10-D-M80059P, ICD-10-D-M80059K, ICD-10-D-M80059G, ICD-10-D-M80059D, ICD-10-D-M80059A, ICD-10-D-M80052S, ICD-10-D-M80052P, ICD-10-D-M80052K, ICD-10-D-M80052G, ICD-10-D-M80052D, ICD-10-D-M80052A, ICD-10-D-M80051S, ICD-10-D-M80051P, ICD-10-D-M80051K, ICD-10-D-M80051G, ICD-10-D-M80051D, ICD-10-D-M80051A, ICD-10-D-M80049S, ICD-10-D-M80049P, ICD-10-D-M80049K, ICD-10-D-M80049G, ICD-10-D-M80049D, ICD-10-D-M80049A, ICD-10-D-M80042S, ICD-10-D-M80042P, ICD-10-D-M80042K, ICD-10-D-M80042G, ICD-10-D-M80042D, ICD-10-D-M80042A, ICD-10-D-M80041S, ICD-10-D-M80041P, ICD-10-D-M80041K, ICD-10-D-M80041G, ICD-10-D-M80041D, ICD-10-D-M80041A, ICD-10-D-M80039S, ICD-10-D-M80039P, ICD-10-D-M80039K, ICD-10-D-M80039G, ICD-10-D-M80039D, ICD-10-D-M80039A, ICD-10-D-M80032S, ICD-10-D-M80032P, ICD-10-D-M80032K, ICD-10-D-M80032G, ICD-10-D-M80032D, ICD-10-D-M80032A, ICD-10-D-M80031S, ICD-10-D-M80031P, ICD-10-D-M80031K, ICD-10-D-M80031G, ICD-10-D-M80031D, ICD-10-D-M80031A, ICD-10-D-M80029S, ICD-10-D-M80029P, ICD-10-D-M80029K, ICD-10-D-M80029G, ICD-10-D-M80029D, ICD-10-D-M80029A, ICD-10-D-M80022S, ICD-10-D-M80022P, ICD-10-D-M80022K, ICD-10-D-M80022G, ICD-10-D-M80022D, ICD-10-D-M80022A, ICD-10-D-M80021S, ICD-10-D-M80021P, ICD-10-D-M80021K, ICD-10-D-M80021G, ICD-10-D-M80021D, ICD-10-D-M80021A, ICD-10-D-M80019S, ICD-10-D-M80019P, ICD-10-D-M80019K, ICD-10-D-M80019G, ICD-10-D-M80019D, ICD-10-D-M80019A, ICD-10-D-M80012S, ICD-10-D-M80012P, ICD-10-D-M80012K, ICD-10-D-M80012G, ICD-10-D-M80012D, ICD-10-D-M80012A, ICD-10-D-M80011S, ICD-10-D-M80011P, ICD-10-D-M80011K, ICD-10-D-M80011G, ICD-10-D-M80011D, ICD-10-D-M80011A, ICD-10-D-M8000XS, ICD-10-D-M8000XP, ICD-10-D-M8000XK, ICD-10-D-M8000XG, ICD-10-D-M8000XD, ICD-10-D-M8000XA |

| **Supplemental Table 4: Billing Codes Used to Define Each Complication** | |
| --- | --- |
| Complication | Billing Codes (ICD-10-CM, ICD-10-PCS, and CPT) |
| Infection | ICD-10-D-B999, ICD-10-D-T84624A, ICD-10-D-T84625A, ICD-10-D-T84629A, ICD-10-D-L089, ICD-10-D-T814, ICD-10-D-T8140XA, ICD-10-D-T8141XA, ICD-10-D-T8142XA, ICD-10-D-T8143XA, ICD-10-D-T8144XA, ICD-10-D-T8149XA, ICD-10-D-L02415, ICD-10-D-L02416, ICD-10-D-L02419, ICD-10-D-L0291, ICD-10-D-L03115, ICD-10-D-L03116, ICD-10-D-L03119, ICD-10-D-L0390, ICD-10-D-M8616, ICD-10-D-M86161, ICD-10-D-M86162, ICD-10-D-M86169, ICD-10-D-M8617, ICD-10-D-M86171, ICD-10-D-M86172, ICD-10-D-M86179, ICD-10-D-M8666, ICD-10-D-M86661, ICD-10-D-M86662, ICD-10-D-M86669, ICD-10-D-M8667, ICD-10-D-M86671, ICD-10-D-M86672, ICD-10-D-M86679, ICD-10-D-M8646, ICD-10-D-M86461, ICD-10-D-M86462, ICD-10-D-M86469, ICD-10-D-M8647, ICD-10-D-M86471, ICD-10-D-M86472, ICD-10-D-M86479, ICD-10-D-M868X6, ICD-10-D-M868X7, ICD-10-D-M8626, ICD-10-D-M86261, ICD-10-D-M86262, ICD-10-D-M86269, ICD-10-D-M8627, ICD-10-D-M86271, ICD-10-D-M86272, ICD-10-D-M86279 |
| Nonunion | ICD-10-D-M80071K, ICD-10-D-M80072K, ICD-10-D-M80079K, ICD-10-D-M80871K, ICD-10-D-M80872K, ICD-10-D-M80879K, ICD-10-D-M84371K, ICD-10-D-M84372K, ICD-10-D-M84373K, ICD-10-D-M84471K, ICD-10-D-M84472K, ICD-10-D-M84473K, ICD-10-D-M84571K, ICD-10-D-M84572K, ICD-10-D-M84573K, ICD-10-D-M84671K, ICD-10-D-M84672K, ICD-10-D-M84673K, ICD-10-D-M84363K, ICD-10-D-M84364K, ICD-10-D-M84369K, ICD-10-D-M84463K, ICD-10-D-M84464K, ICD-10-D-M84469K, ICD-10-D-M84563K, ICD-10-D-M84564K, ICD-10-D-M84569K, ICD-10-D-M84663K, ICD-10-D-M84664K, ICD-10-D-M84669K, ICD-10-D-S82401K, ICD-10-D-S82401M, ICD-10-D-S82401N, ICD-10-D-S82402K, ICD-10-D-S82402M, ICD-10-D-S82402N, ICD-10-D-S82409K, ICD-10-D-S82409M, ICD-10-D-S82409N, ICD-10-D-S82421K, ICD-10-D-S82421M, ICD-10-D-S82421N, ICD-10-D-S82422K, ICD-10-D-S82422M, ICD-10-D-S82422N, ICD-10-D-S82423K, ICD-10-D-S82423M, ICD-10-D-S82424K, ICD-10-D-S82424M, ICD-10-D-S82424N, ICD-10-D-S82425K, ICD-10-D-S82425M, ICD-10-D-S82425N, ICD-10-D-S82426K, ICD-10-D-S82431K, ICD-10-D-S82431M, ICD-10-D-S82431N, ICD-10-D-S82432K, ICD-10-D-S82432M, ICD-10-D-S82432N, ICD-10-D-S82433K, ICD-10-D-S82434K, ICD-10-D-S82434M, ICD-10-D-S82435K, ICD-10-D-S82435M, ICD-10-D-S82436K, ICD-10-D-S82436M, ICD-10-D-S82441K, ICD-10-D-S82441M, ICD-10-D-S82441N, ICD-10-D-S82442K, ICD-10-D-S82442M, ICD-10-D-S82442N, ICD-10-D-S82443K, ICD-10-D-S82444K, ICD-10-D-S82444M, ICD-10-D-S82444N, ICD-10-D-S82445K, ICD-10-D-S82445M, ICD-10-D-S82445N, ICD-10-D-S82446K, ICD-10-D-S82446M, ICD-10-D-S82451K, ICD-10-D-S82451M, ICD-10-D-S82451N, ICD-10-D-S82452K, ICD-10-D-S82452M, ICD-10-D-S82452N, ICD-10-D-S82453K, ICD-10-D-S82453M, ICD-10-D-S82454K, ICD-10-D-S82454M, ICD-10-D-S82455K, ICD-10-D-S82455M, ICD-10-D-S82455N, ICD-10-D-S82456K, ICD-10-D-S82456M, ICD-10-D-S82461K, ICD-10-D-S82461M, ICD-10-D-S82461N, ICD-10-D-S82462K, ICD-10-D-S82462M, ICD-10-D-S82462N, ICD-10-D-S82463K, ICD-10-D-S82464K, ICD-10-D-S82465K, ICD-10-D-S82466K, ICD-10-D-S82466N, ICD-10-D-S82491K, ICD-10-D-S82491M, ICD-10-D-S82491N, ICD-10-D-S82492K, ICD-10-D-S82492M, ICD-10-D-S82492N, ICD-10-D-S82499K, ICD-10-D-S8261XK, ICD-10-D-S8261XM, ICD-10-D-S8261XN, ICD-10-D-S8262XK, ICD-10-D-S8262XM, ICD-10-D-S8262XN, ICD-10-D-S8263XK, ICD-10-D-S8263XM, ICD-10-D-S8263XN, ICD-10-D-S8264XK, ICD-10-D-S8264XM, ICD-10-D-S8264XN, ICD-10-D-S8265XK, ICD-10-D-S8265XM, ICD-10-D-S8265XN, ICD-10-D-S8266XK, ICD-10-D-S8266XM, ICD-10-D-S8266XN, ICD-10-D-S82821K, ICD-10-D-S82822K, ICD-10-D-S82829K, ICD-10-D-S82831K, ICD-10-D-S82831M, ICD-10-D-S82831N, ICD-10-D-S82832K, ICD-10-D-S82832M, ICD-10-D-S82832N, ICD-10-D-S82839K, ICD-10-D-S82839M, ICD-10-D-S89301K, ICD-10-D-S89302K, ICD-10-D-S89309K, ICD-10-D-S89311K, ICD-10-D-S89312K, ICD-10-D-S89321K, ICD-10-D-S89322K, ICD-10-D-S89329K, ICD-10-D-S89391K, ICD-10-D-S89392K, ICD-10-D-S89399K, ICD-10-D-M80061K, ICD-10-D-M80062K, ICD-10-D-M80069K, ICD-10-D-M80861K, ICD-10-D-M80862K, ICD-10-D-M80869K, ICD-10-D-S82841K, ICD-10-D-S82841M, ICD-10-D-S82841N, ICD-10-D-S82842K, ICD-10-D-S82842M, ICD-10-D-S82842N, ICD-10-D-S82843K, ICD-10-D-S82843M, ICD-10-D-S82843N, ICD-10-D-S82844K, ICD-10-D-S82844M, ICD-10-D-S82844N, ICD-10-D-S82845K, ICD-10-D-S82845M, ICD-10-D-S82845N, ICD-10-D-S82846K, ICD-10-D-S82846M, ICD-10-D-S82846N, ICD-10-D-S82851K, ICD-10-D-S82851M, ICD-10-D-S82851N, ICD-10-D-S82852K, ICD-10-D-S82852M, ICD-10-D-S82852N, ICD-10-D-S82853K, ICD-10-D-S82853M, ICD-10-D-S82853N, ICD-10-D-S82854K, ICD-10-D-S82854M, ICD-10-D-S82854N, ICD-10-D-S82855K, ICD-10-D-S82855M, ICD-10-D-S82855N, ICD-10-D-S82856K, ICD-10-D-S82856M, ICD-10-D-S82856N, ICD-10-D-S82891K, ICD-10-D-S82891M, ICD-10-D-S82891N, ICD-10-D-S82892K, ICD-10-D-S82892M, ICD-10-D-S82892N, ICD-10-D-S82899K, ICD-10-D-S82899M, ICD-10-D-S82899N, ICD-10-D-S8290XK, ICD-10-D-S8290XM, ICD-10-D-S8290XN, ICD-10-D-S8291XK, ICD-10-D-S8291XM, ICD-10-D-S8291XN, ICD-10-D-S8292XK, ICD-10-D-S8292XM, ICD-10-D-S8292XN |
| Malunion | ICD-10-D-M80071P, ICD-10-D-M80072P, ICD-10-D-M80079P, ICD-10-D-M80871P, ICD-10-D-M80872P, ICD-10-D-M80879P, ICD-10-D-M84371P, ICD-10-D-M84372P, ICD-10-D-M84373P, ICD-10-D-M84471P, ICD-10-D-M84472P, ICD-10-D-M84473P, ICD-10-D-M84571P, ICD-10-D-M84572P, ICD-10-D-M84573P, ICD-10-D-M84671P, ICD-10-D-M84672P, ICD-10-D-M84673P, ICD-10-D-M84363P, ICD-10-D-M84364P, ICD-10-D-M84369P, ICD-10-D-M84463P, ICD-10-D-M84464P, ICD-10-D-M84469P, ICD-10-D-M84563P, ICD-10-D-M84564P, ICD-10-D-M84569P, ICD-10-D-M84663P, ICD-10-D-M84664P, ICD-10-D-M84669P, ICD-10-D-S82401P, ICD-10-D-S82401Q, ICD-10-D-S82401R, ICD-10-D-S82402P, ICD-10-D-S82402Q, ICD-10-D-S82402R, ICD-10-D-S82409P, ICD-10-D-S82409Q, ICD-10-D-S82409R, ICD-10-D-S82421P, ICD-10-D-S82421Q, ICD-10-D-S82422P, ICD-10-D-S82422Q, ICD-10-D-S82422R, ICD-10-D-S82423P, ICD-10-D-S82424P, ICD-10-D-S82424Q, ICD-10-D-S82424R, ICD-10-D-S82425P, ICD-10-D-S82425Q, ICD-10-D-S82426Q, ICD-10-D-S82431P, ICD-10-D-S82431Q, ICD-10-D-S82432P, ICD-10-D-S82432Q, ICD-10-D-S82432R, ICD-10-D-S82433P, ICD-10-D-S82434P, ICD-10-D-S82435P, ICD-10-D-S82436P, ICD-10-D-S82441P, ICD-10-D-S82442P, ICD-10-D-S82442Q, ICD-10-D-S82444P, ICD-10-D-S82445P, ICD-10-D-S82446P, ICD-10-D-S82451P, ICD-10-D-S82451Q, ICD-10-D-S82451R, ICD-10-D-S82452P, ICD-10-D-S82452Q, ICD-10-D-S82452R, ICD-10-D-S82453P, ICD-10-D-S82453R, ICD-10-D-S82454P, ICD-10-D-S82454Q, ICD-10-D-S82454R, ICD-10-D-S82455P, ICD-10-D-S82456Q, ICD-10-D-S82456R, ICD-10-D-S82461P, ICD-10-D-S82461Q, ICD-10-D-S82461R, ICD-10-D-S82462P, ICD-10-D-S82462R, ICD-10-D-S82463P, ICD-10-D-S82464P, ICD-10-D-S82464Q, ICD-10-D-S82465P, ICD-10-D-S82465R, ICD-10-D-S82466P, ICD-10-D-S82491P, ICD-10-D-S82491Q, ICD-10-D-S82491R, ICD-10-D-S82492P, ICD-10-D-S82492Q, ICD-10-D-S82492R, ICD-10-D-S82499P, ICD-10-D-S82499Q, ICD-10-D-S8261XP, ICD-10-D-S8261XQ, ICD-10-D-S8261XR, ICD-10-D-S8262XP, ICD-10-D-S8262XQ, ICD-10-D-S8262XR, ICD-10-D-S8263XP, ICD-10-D-S8263XQ, ICD-10-D-S8263XR, ICD-10-D-S8264XP, ICD-10-D-S8264XQ, ICD-10-D-S8264XR, ICD-10-D-S8265XP, ICD-10-D-S8265XQ, ICD-10-D-S8265XR, ICD-10-D-S8266XP, ICD-10-D-S8266XQ, ICD-10-D-S82821P, ICD-10-D-S82822P, ICD-10-D-S82829P, ICD-10-D-S82831P, ICD-10-D-S82831Q, ICD-10-D-S82831R, ICD-10-D-S82832P, ICD-10-D-S82832Q, ICD-10-D-S82832R, ICD-10-D-S82839P, ICD-10-D-S82839Q, ICD-10-D-S82839R, ICD-10-D-S89301P, ICD-10-D-S89302P, ICD-10-D-S89311P, ICD-10-D-S89312P, ICD-10-D-S89319P, ICD-10-D-S89321P, ICD-10-D-S89322P, ICD-10-D-S89329P, ICD-10-D-S89391P, ICD-10-D-S89392P, ICD-10-D-S89399P, ICD-10-D-M80061P, ICD-10-D-M80062P, ICD-10-D-M80069P, ICD-10-D-M80861P, ICD-10-D-M80862P, ICD-10-D-M80869P, ICD-10-D-S82841P, ICD-10-D-S82841Q, ICD-10-D-S82841R, ICD-10-D-S82842P, ICD-10-D-S82842Q, ICD-10-D-S82842R, ICD-10-D-S82843P, ICD-10-D-S82843Q, ICD-10-D-S82843R, ICD-10-D-S82844P, ICD-10-D-S82844Q, ICD-10-D-S82844R, ICD-10-D-S82845P, ICD-10-D-S82845Q, ICD-10-D-S82845R, ICD-10-D-S82846P, ICD-10-D-S82846Q, ICD-10-D-S82846R, ICD-10-D-S82851P, ICD-10-D-S82851Q, ICD-10-D-S82851R, ICD-10-D-S82852P, ICD-10-D-S82852Q, ICD-10-D-S82852R, ICD-10-D-S82853P, ICD-10-D-S82853Q, ICD-10-D-S82853R, ICD-10-D-S82854P, ICD-10-D-S82854Q, ICD-10-D-S82854R, ICD-10-D-S82855P, ICD-10-D-S82855Q, ICD-10-D-S82855R, ICD-10-D-S82856P, ICD-10-D-S82856Q, ICD-10-D-S82856R, ICD-10-D-S82891P, ICD-10-D-S82891Q, ICD-10-D-S82891R, ICD-10-D-S82892P, ICD-10-D-S82892Q, ICD-10-D-S82892R, ICD-10-D-S82899P, ICD-10-D-S82899Q, ICD-10-D-S82899R, ICD-10-D-S8290XP, ICD-10-D-S8290XQ, ICD-10-D-S8290XR, ICD-10-D-S8291XP, ICD-10-D-S8291XQ, ICD-10-D-S8291XR, ICD-10-D-S8292XP, ICD-10-D-S8292XQ, ICD-10-D-S8292XR |
| Radiology Codes (For confirmation of malunion/nonunion) | CPT-73590, CPT-73600, CPT-73610, CPT-73700, ICD-10-P-BQ0DZZZ, ICD-10-P-BQ0FZZZ, ICD-10-P-BW0CZZZ, ICD-10-P-BQ0GZZZ, ICD-10-P-BQ0HZZZ, ICD-10-P-BQ2DZZZ, ICD-10-P-BQ2FZZZ, ICD-10-P-BQ2GZZZ, ICD-10-P-BQ2HZZZ, ICD-10-P-BQ2RZZZ, ICD-10-P-BQ2SZZZ |
| Revision | ICD-10-P-0QWJ04Z, ICD-10-P-0QWK04Z, ICD-10-P-0QWJ34Z, ICD-10-P-0QWK34Z, ICD-10-P-0QWJ44Z, ICD-10-P-0QWK44Z, ICD-10-P-0QWJX4Z, ICD-10-P-0QWKX4Z, CPT-27726, ICD-10-P-0QHJ04Z, ICD-10-P-0QHK04Z, ICD-10-P-0QHJ34Z, ICD-10-P-0QHK34Z, ICD-10-P-0QHJ44Z, ICD-10-P-0QHK44Z, ICD-10-P-0QSJ04Z, ICD-10-P-0QSK04Z, ICD-10-P-0QSJ34Z, ICD-10-P-0QSK34Z, ICD-10-P-0QSJ44Z, ICD-10-P-0QSK44Z, ICD-10-P-0QHJ06Z, ICD-10-P-0QHK06Z, ICD-10-P-0QHJ36Z, ICD-10-P-0QHK36Z, ICD-10-P-0QHJ46Z, ICD-10-P-0QHK46Z, ICD-10-P-0QSJ06Z, ICD-10-P-0QSK06Z, ICD-10-P-0QSJ36Z, ICD-10-P-0QSK36Z, ICD-10-P-0QSJ46Z, ICD-10-P-0QSK46Z, ICD-10-P-0QQJ0ZZ, ICD-10-P-0QQK0ZZ, ICD-10-P-0QQJ3ZZ, ICD-10-P-0QQK3ZZ, ICD-10-P-0QQJ4ZZ, ICD-10-P-0QQK4ZZ, ICD-10-P-0QSJ0ZZ, ICD-10-P-0QSK0ZZ, ICD-10-P-0QSJ3ZZ, ICD-10-P-0QSK3ZZ, ICD-10-P-0QSJ4ZZ, ICD-10-P-0QSK4ZZ, CPT-27732, CPT-27734, CPT-27740, CPT-27742, CPT-27784, CPT-27792, CPT-27814, CPT-27822, CPT-27823, CPT-27826, CPT-27828, CPT-27829, CPT-27846, CPT-27848 |
| Hardware Removal | ICD-10-P-0QPJ04Z, ICD-10-P-0QPJ34Z, ICD-10-P-0QPJX4Z, ICD-10-P-0QPK04Z, ICD-10-P-0QPK34Z, ICD-10-P-0QPK44Z, ICD-10-P-0QPKX4Z, ICD-10-P-0QPJ44Z, CPT-27704, CPT-20680 |
| Pulmonary Embolism | ICD-10-D-I26:ICD-10-D-I269 |
| Deep Vein Thrombosis | ICD-10-D-I26:ICD-10-D-I2699 |

| **Supplemental Table 5.** Matched Demographics of Patients with Distal Fibular Fractures and At Least 12 Months of Follow-up | | | |
| --- | --- | --- | --- |
| **Characteristic** | **IMF (N = 316)***^1^* | **PF (N = 1254)***^1^* | **p-value** |
| Age Mean ± SD | 60.51 ± 15.70 | 60.48 ± 15.65 (18-82) | 0.976 |
| Age Median (IQR) | 63 (52-74) | 63 (51-73) |  |
| Age |  |  |  |
| 18-19 | 1 (0.3%) | 2 (0.2%) | 0.568 |
| 20-29 | 17 (5.4%) | 67 (5.3%) | 0.979 |
| 30-39 | 23 (0%) | 92 (7.3%) | 0.972 |
| 40-49 | 31 (9.8%) | 122 (9.7%) | 0.965 |
| 50-59 | 57 (18.0%) | 226 (18.0%) | 0.995 |
| 60-69 | 82 (25.9%) | 327 (26.1%) | 0.963 |
| 70+ | 105 (33.2%) | 418 (33.3%) | 0.972 |
| Female | 217 (68.7%) | 865 (69.0%) | 0.915 |
| ECI Score | 7.07 ± 4.81 | 6.75 ± 4.39 | 0.256 |
| Diabetes | 150 (47.5%) | 595 (47.4%) | 0.995 |
| Diabetes (Complicated) | 92 (29.1%) | 359 (28.6%) | 0.865 |
| Tobacco Use | 109 (34.5%) | 431 (34.4%) | 0.968 |
| Obesity | 99 (31.3%) | 390 (31.1%) | 0.937 |
| Chronic Kidney Disease | 71 (22.5%) | 278 (22.2 %) | 0.909 |
| High Energy Fracture | 187 (59.2%) | 743 (59.3%) | 0.976 |
| *^1^*n (%)  **IMF:** Intramedullary Fixation  **PF:** Plate Fixation  **ECI:** Elixhauser Comorbidity Index | | | |
